# CDP-ribitol prodrug treatment ameliorates *ISPD-*deficient muscular dystrophy mouse model

**DOI:** 10.1038/s41467-022-29473-4

**Published:** 2022-04-14

**Authors:** Hideki Tokuoka, Rieko Imae, Hitomi Nakashima, Hiroshi Manya, Chiaki Masuda, Shunsuke Hoshino, Kazuhiro Kobayashi, Dirk J. Lefeber, Riki Matsumoto, Takashi Okada, Tamao Endo, Motoi Kanagawa, Tatsushi Toda

**Affiliations:** 1 Division of Molecular Brain Science, Kobe University Graduate School of Medicine, Kobe, Hyogo 650-0017 Japan; 2 Division of Neurology, Kobe University Graduate School of Medicine, Kobe, Hyogo 650-0017 Japan; 3 Molecular Glycobiology, Research Team for Mechanism of Aging, Tokyo Metropolitan Geriatric Hospital and Institute of Gerontology, Itabashi-ku, Tokyo, 173-0015 Japan; 4 Department of Biochemistry and Molecular Biology, Nippon Medical School, Bunkyo-ku, Tokyo, 113-8602 Japan; 5 Department of Neurology, Donders Institute for Brain, Cognition and Behavior, Radboud University Medical Center, Nijmegen, the Netherlands; Translational Metabolic Laboratory, Department of Laboratory Medicine, Radboud Institute for Molecular Life Sciences, Radboud University Medical Center, 6525 GA Nijmegen, The Netherlands; 6 Division of Molecular and Medical Genetics, Center for Gene and Cell Therapy, The Institute of Medical Science, The University of Tokyo, Minato-ku, Tokyo, 108-8639 Japan; 7 Department of Cell Biology and Molecular Medicine, Ehime University Graduate School of Medicine, Toon, Ehime 791-0295 Japan; 8 Department of Neurology, Graduate School of Medicine, The University of Tokyo, Bunkyo-ku, Tokyo, 113-8655 Japan

**Keywords:** Glycobiology, Drug discovery, Neuromuscular disease

## Abstract

Ribitol-phosphate modification is crucial for the functional maturation of α-dystroglycan. Its dysfunction is associated with muscular dystrophy, cardiomyopathy, and central nervous system abnormalities; however, no effective treatments are currently available for diseases caused by ribitol-phosphate defects. In this study, we demonstrate that prodrug treatments can ameliorate muscular dystrophy caused by defects in *isoprenoid synthase domain containing* (*ISPD*), which encodes an enzyme that synthesizes CDP-ribitol, a donor substrate for ribitol-phosphate modification. We generated skeletal muscle-selective *Ispd* conditional knockout mice, leading to a pathogenic reduction in CDP-ribitol levels, abnormal glycosylation of α-dystroglycan, and severe muscular dystrophy. Adeno-associated virus-mediated gene replacement experiments suggested that the recovery of CDP-ribitol levels rescues the ISPD-deficient pathology. As a prodrug treatment strategy, we developed a series of membrane-permeable CDP-ribitol derivatives, among which tetraacetylated CDP-ribitol ameliorated the dystrophic pathology. In addition, the prodrug successfully rescued abnormal α-dystroglycan glycosylation in patient fibroblasts. Consequently, our findings provide proof-of-concept for supplementation therapy with CDP-ribitol and could accelerate the development of therapeutic agents for muscular dystrophy and other diseases caused by glycosylation defects.

## Abstract (web summary)

Ribitol-phospate modification is essential for the function of α-dystroglycan, and mutations in ISPD, an enzyme that synthesizes the the ribitol-phosphate donor CDP-ribitol, cause muscular dystrophy. Here, the authors show that recovery of CDP-ribitol levels, either via AAV-mediated gene therapy or prodrug treatment, rescues dystroglycan function and pathology in a mouse model.

## Introduction

Muscular dystrophies are a heterogeneous group of genetic disorders characterized by the progressive weakening of skeletal muscle. Several types of muscular dystrophy are caused by mutations in components of the dystrophin-glycoprotein complex (DGC), which forms a structural link between the basement membrane and the actin cytoskeleton that is essential for maintaining the physical stability of muscle. Thus, genetic disruptions in the DGC lead to pathological muscle cell weakness^1,2^. A central component of the DGC is dystroglycan (DG), which comprises the highly glycosylated alpha-DG (α-DG) and the transmembrane protein beta-DG (β-DG) that directly connects the basement membrane and the dystrophin-cytoskeleton across the plasma membrane. In particular, α-DG binds to extracellular matrix proteins, such as laminins, and glycosylation is essential for ligand-binding activity. β-DG binds to dystrophin underneath the cell membrane.

Abnormal glycosylation caused by genetic mutations is associated with a group of muscular dystrophies known as dystroglycanopathies^3,4^. At least 18 genes are currently known to cause dystroglycanopathy, most of which encode enzymes responsible for α-DG glycosylation^5,6^. α-DG sugar chains contain *O-*mannose (Man)-type glycans, namely Core M1 and Core M3 glycans^7,8^. The terminal end of Core M3 glycans is formed of repeating units consisting of glucuronic acid (GlcA) and xylose (Xyl), which are called matriglycan that serves as a ligand-binding domain^9,10^. A tandem ribitol-phosphate (RboP) moiety is present between matriglycan and the Core M3 glycan^11^. Because this linker is required for matriglycan modification, loss of the RboP modification also results in dystroglycanopathy.

Three dystroglycanopathy genes have been reported to encode key enzymes required for tandem RboP synthesis, namely *fukutin*^12^*, fukutin-related protein (FKRP)*^13^, and *isoprenoid synthase domain-containing protein (ISPD)*, also known as *CRPPA*^14,15^ (Supplementary Fig. 1). Fukutin and FKRP are RboP transferases that sequentially transfer the RboP moiety from CDP-ribitol (CDP-Rbo) onto Core M3^11^, whereas *ISPD* encodes a D-ribitol-5-phosphate cytidylyltransferase that synthesizes CDP-Rbo from CTP and D-ribitol-5-phosphate^11,16,17^. Since CDP-Rbo serves as a donor substrate for fukutin and FKRP, defects in ISPD affect the fukutin- and FKRP-dependent RboP modification of α-DG.

*ISPD* mutations were originally identified in patients with Walker-Warburg syndrome, a severe congenital muscular dystrophy, as well as limb-girdle muscular dystrophy (LGMD) 2U^14,15,18^. Several studies have suggested that ~10 % of severe dystroglycanopathy cases are associated with *ISPD* mutations^14,15,19^. A population study suggested that ~4 % of all patients with pediatric-onset LGMD had *ISPD* mutations^20^. Although the enzyme activity of ISPD has been revealed in vitro, there is a lack of direct evidence that ISPD is responsible for CDP-Rbo production in vivo and no treatments for ISPD-deficient muscular dystrophy are currently available.

Previously, we reported that CDP-Rbo supplementation can effectively restore α-DG glycosylation in ISPD-deficient cultured cells;^11^ however, the efficacy of CDP-Rbo supplementation therapy in vivo has not yet been demonstrated. Therefore, we generated skeletal muscle-specific *Ispd* conditional knockout (cKO) mice and showed that the lack of CDP-Rbo production caused by *Ispd*-deficiency leads to muscular dystrophy. We then performed gene replacement studies to demonstrate that ISPD-deficient muscular dystrophy can be treated even after disease onset. Our data provide evidence that CDP-Rbo supplementation using prodrugs can restore α-DG glycosylation and ameliorate the dystrophic pathology in a mouse model of ISPD-deficient muscular dystrophy.

## Results

### Generation and characterization of skeletal muscle-selective *Ispd* cKO mice

To generate *Ispd* cKO mice, we crossed flox *Ispd* mice (*Ispd*^*lox/lox*^)^21^ with Myf5-Cre knock-in (KI) mice expressing Cre recombinase via an endogenous *Myf5* promoter^22^. Myf5-Cre KI mice are widely used to generate skeletal muscle-selective cKO mice^23,24^ as the myogenic regulatory factor Myf5 is expressed in muscle precursor cells during early skeletal muscle development and adult muscle regeneration^25^, allowing the complete loss of the targeted floxed gene in skeletal muscle. Our breeding strategy produced three genotypes (see Methods): [*Ispd*^*lox/lox*^: *Myf5-Cre*^*KI*^ (-)], [*Ispd*^*lox/+*^: *Myf5-Cre*^*KI*^ (+)], and [*Ispd*^*lox/lox*^: *Myf5-Cre*^*KI*^ (+)] mice, which were used as wild-type controls (WT), heterozygous controls (Het), and Myf5-*Ispd*-cKO (cKO), respectively. Reverse transcription (RT) PCR analysis of *Ispd* mRNA expression in skeletal muscle confirmed genotype-dependent decreases in the *Ispd* expression (Supplementary Fig. 2a).

**Fig. 1:**
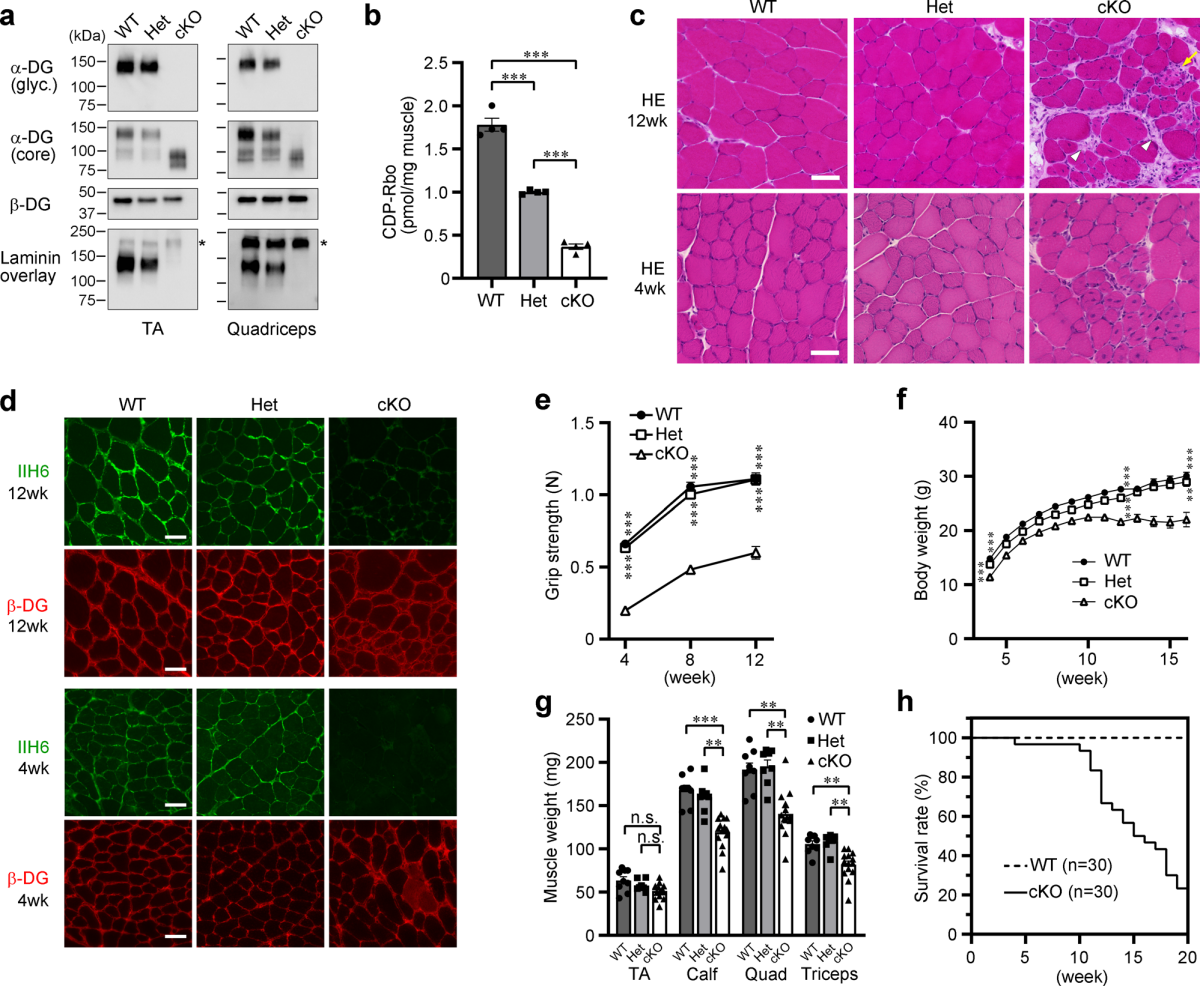
Biochemical and pathological characterization of skeletal muscle from Myf5-*Ispd*-cKO mice. **a** Glycosylation and laminin-binding activity of α-DG from the skeletal muscle of WT, Het, and cKO mice (12-weeks-old) analyzed using western blotting and laminin overlay assays with antibodies against matriglycan (glyc., IIH6) and core protein (3D7). β-DG was used as a loading control. *, endogenous laminin. **b** Endogenous CDP-Rbo concentration in skeletal muscle tissue extracts (TA) detected using LC-MS. Data were analyzed using ANOVA with Tukey’s post hoc test (*p* < 0.0001 for each comparison. *n* = 4). **c**, **d** Representative HE staining images (**c**) and DG immunofluorescence analysis (**d**) in the skeletal muscle (TA) of 4- and 12-week-old mice. Arrow, necrotic fiber; arrow head, fibrous connective tissue. **e**, **f** Temporal changes in the grip strength (**e**) and body weight (**f**) of cKO, Het, and WT mice. (**e**: *p* < 0.0001 for each cKO vs. WT and cKO vs. Het comparison. WT, *n* = 46, 41, and 25; Het, *n* = 41, 40, and 31; a*n*d cKO, *n* = 54, 53, and 30 at 4, 8, and 12 weeks, respectively. **f**: *p* < 0.0001, *p* < 0.0001, and *p* = 0.0001 for cKO vs. WT; *p* = 0.0004, *p* < 0.0001, and *p* = 0.0018 for cKO vs. Het at 4, 12, and 16 weeks, respectively. WT, *n* = 50, 30, and 17; Het, *n* = 45, 33, and 19; and cKO, *n* = 62, 39, and 12 at 4, 12, and 16 weeks, respectively). **g** Muscle weight in 12-week-old mice (Calf: *p* < 0.0001, cKO vs. WT; *p* = 0.005, cKO vs. Het. Quad: *p* = 0.0031, cKO vs. WT; *p* = 0.001, cKO vs. Het. Triceps: *p* = 0.0028, cKO vs. WT; *p* = 0.0012, cKO vs. Het. WT, *n* = 9; Het, *n* = 8; cKO, *n* = 14). **h** Survival of Myf5-*Ispd*-cKO mice (*n* = 30). All data represent the mean ± SEM. Data were analyzed usi*n*g the Kruskal-Wallis ANOVA test followed by Dunn’s multiple comparison (**e**–**h**). **p < 0.01, ***p < 0.001. Scale bars, 50 μm. TA, tibialis anterior. Quad, quadriceps.

To examine the glycosylation status of α-DG in Myf5-*Ispd*-cKO mice, we enriched α-DG from skeletal muscle extracts using wheat germ agglutinin (WGA)-beads and then analyzed the DG-enriched preparations using western blotting with antibodies against the laminin-binding glycan moiety (matriglycan) (IIH6), α-DG core protein (3D7), and β-DG, as well as using a laminin overlay binding assay (Fig. 1a). We observed abnormal α-DG glycosylation in the skeletal muscle of Myf5-*Ispd-*cKO mice, as indicated by the loss of immunoreactivity against the IIH6 antibody (glyc.), a decrease in the molecular weight of α-DG (core), and a decrease in laminin-binding activity.

Since *Ispd* encodes D-ribitol-5-phosphate cytidylyltransferase, an enzyme that synthesizes CDP-Rbo^11,17^, we examined CDP-Rbo levels in skeletal muscle. Quantitative analysis of CDP-Rbo using liquid chromatography (LC)/ mass spectrometry (MS) revealed that WT skeletal muscle contained ~1.7 pmol/mg muscle, whereas cKO contained less than 0.3 pmol/mg skeletal muscle (Fig. 1b). Het skeletal muscle contained an intermediate concentration (~1.0 pmol/mg skeletal muscle), indicating that CDP-Rbo levels are associated with genotype. CDP-Rbo serves as the donor substrate for the RboP transferases fukutin and FKRP; therefore, reduced cellular CDP-Rbo levels are expected to result in defective α-DG RboP-modification, abnormal glycosylation, and reduced ligand-binding activity. Importantly, abnormal glycosylation was not observed in the Het controls, suggesting that intermediate CDP-Rbo levels (~1.0 pmol/mg skeletal muscle) are sufficient to prevent abnormal α-DG glycosylation. Together, these data constitute the first in vivo evidence that Ispd acts as a CDP-Rbo synthase required for α-DG glycosylation in skeletal muscle.

Next, we examined the skeletal muscle phenotype of Myf5-*Ispd*-cKO mice. Hematoxylin and eosin (HE) staining showed that the 12-week-old cKO mice displayed signs of muscular dystrophy, such as necrotic and regenerating fibers, central nucleation, and fibrous connective tissue infiltration, which were also observed mildly in 4-week-old cKO mice (Fig. 1c and Supplementary Fig. 2b). Immunofluorescence analysis indicated that IIH6 antibody immunoreactivity was reduced in both 4- and 12-week-old cKO mice (Fig. 1d), suggesting that the pathological changes observed in cKO mice began at a young age. We also examined changes in grip strength, body weight, muscle weight, and serum creatine kinase levels in the mouse models, finding that the grip strength of cKO mice was considerably weaker than that of the Het and WT mice at any age (4, 8, and 12 weeks) (Fig. 1e). In addition, the body weight of cKO mice increased significantly slower at a young age and stopped entirely at 10 weeks (Fig. 1f), while muscle weight (calf, quadriceps, and triceps) was significantly lower in the cKO mice than in the control mice at 12 weeks of age (Fig. 1g). Furthermore, some cKO mice died after 10-weeks of age and most had died by 20-weeks of age (Fig. 1h).

**Fig. 2:**
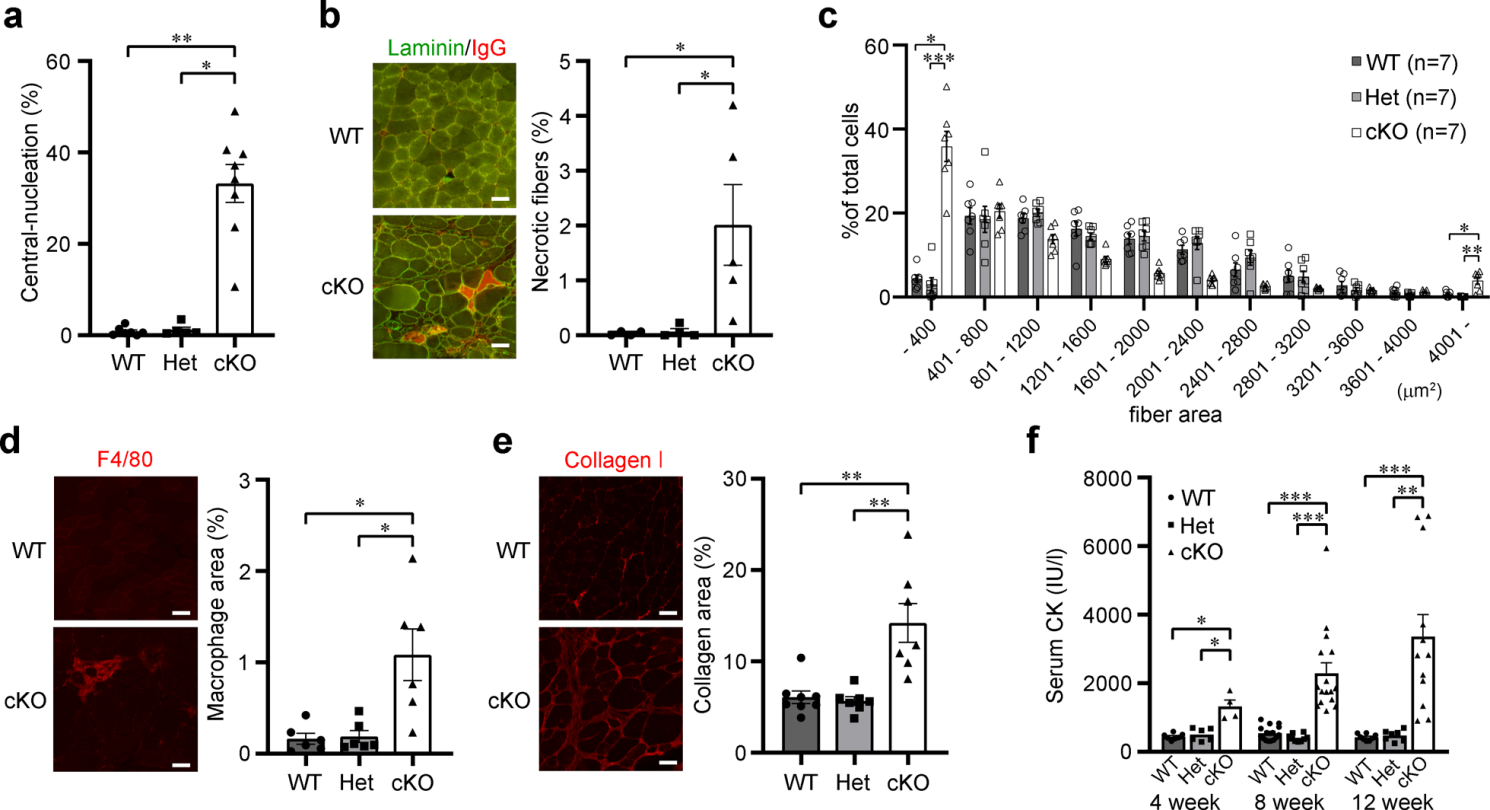
Quantitative analysis of skeletal muscle pathology in Myf5-*Ispd*-cKO mice. **a** Quantification of myofibers with centrally located nuclei (*p* = 0.0015, cKO vs. WT; *p* = 0.021, cKO vs. Het. WT, *n* = 7; Het, *n* = 5; cKO, *n* = 8). **b** Immunofluorescence and quantitative analysis of necrotic fibers. Percentage of muscle fibers (TA) with intracellular IgG signals (*p* = 0.035, cKO vs. WT; *p* = 0.035, cKO vs. Het. WT, *n* = 4; Het, *n* = 4; cKO, *n* = 5). **c** Quantitative analysis of myofiber size variation (*p* = 0.029, cKO vs. WT; *p* = 0.0005, cKO vs. Het < 400 μm^2^; *p* = 0.047, cKO vs. WT; *p* = 0.001, cKO vs. Het > 4000 μm^2^; *n* = 7 per genotype). **d**, **e** Immunofluorescence and quantitative analysis of macrophage infiltration (**d**) and connective tissue area (**e**). Quantitative analysis of muscle sections (TA) stained with antibodies against F4/80 (macrophage marker) or collagen I (connective tissue marker; **d**: *p* = 0.018, cKO vs. WT; *p* = 0.045, cKO vs. Het. *n* = 6 per genotype. **e**: *p* = 0.009, cKO vs. WT, *p* = 0.003, cKO vs. Het. WT, *n* = 8; Het, *n* = 7*;* cKO, *n* = 7). **f** Temporal changes in serum CK activity (*p* = 0.017, cKO vs. WT; *p* = 0.037, cKO vs. Het at 4 weeks. *p* < 0.0001 for both cKO vs. WT and cKO vs. Het at 8 weeks. *p* = 0.0002, cKO vs. WT; *p* = 0.003, cKO vs. Het at 12 weeks. WT, *n* = 7, 15, and 8; Het, *n* = 6, 9, and 7; and cKO, *n* = 4, 16, and 12 at 4, 8, and 12 weeks, respectively). All data represent the mean ± SEM. Data were analyzed using the Kruskal-Wallis ANOVA test followed by Dunn’s multiple comparisons. *p < 0.05, **p < 0.01, and ***p < 0.001. Scale bars, 50 μm. TA, tibialis anterior.

In addition, we quantitatively examined the pathological changes in cKO mice. The proportion of regenerating myofibers with centrally located nuclei and necrotic myofibers was dramatically higher in 12-week-old cKO mice than in WT and Het mice (Fig. 2a, b). Moreover, the 12-week-old cKO mice displayed advanced pathological changes, such as fiber size variation, macrophage infiltration, and fibrosis (Fig. 2c–e). Serum creatine kinase (CK) levels were also significantly increased in cKO mice at all ages examined (Fig. 2f). Together, these data clearly demonstrate that Myf5-*Ispd*-cKO mice have a muscular dystrophic phenotype.

A small number of Myf5-*Ispd*-cKO mice showed incomplete loss of α-DG glycosylation and a much milder muscle phenotype (Supplementary Fig. 2c, d), consistent with previously reported variation in the individual phenotypes of other models of dystroglycanopathy generated using Myf5-Cre KI mice^23,24^. The levels of α-DG glycosylation in the skeletal muscle of milder cKO mice were less than 10% of those in WT mice (Supplementary Fig. 2c). This variation may be due to the amount, activity, or circumstances of Cre recombinase affecting the efficacy of Cre-recombination, or the presence of an Myf5-independent lineage^26,27^. Importantly, we were able to distinguish Myf5-*Ispd*-cKO mice with a milder phenotype by measuring grip strength at 4 weeks of age; thus, only Myf5-*Ispd*-cKO mice with a grip strength of <0.4 N at 4 weeks were used for the pathological analyses and subsequent therapeutic experiments.

### Gene replacement ameliorates the dystrophic phenotype of Myf5-*Ispd*-cKO mice

**Fig. 3:**
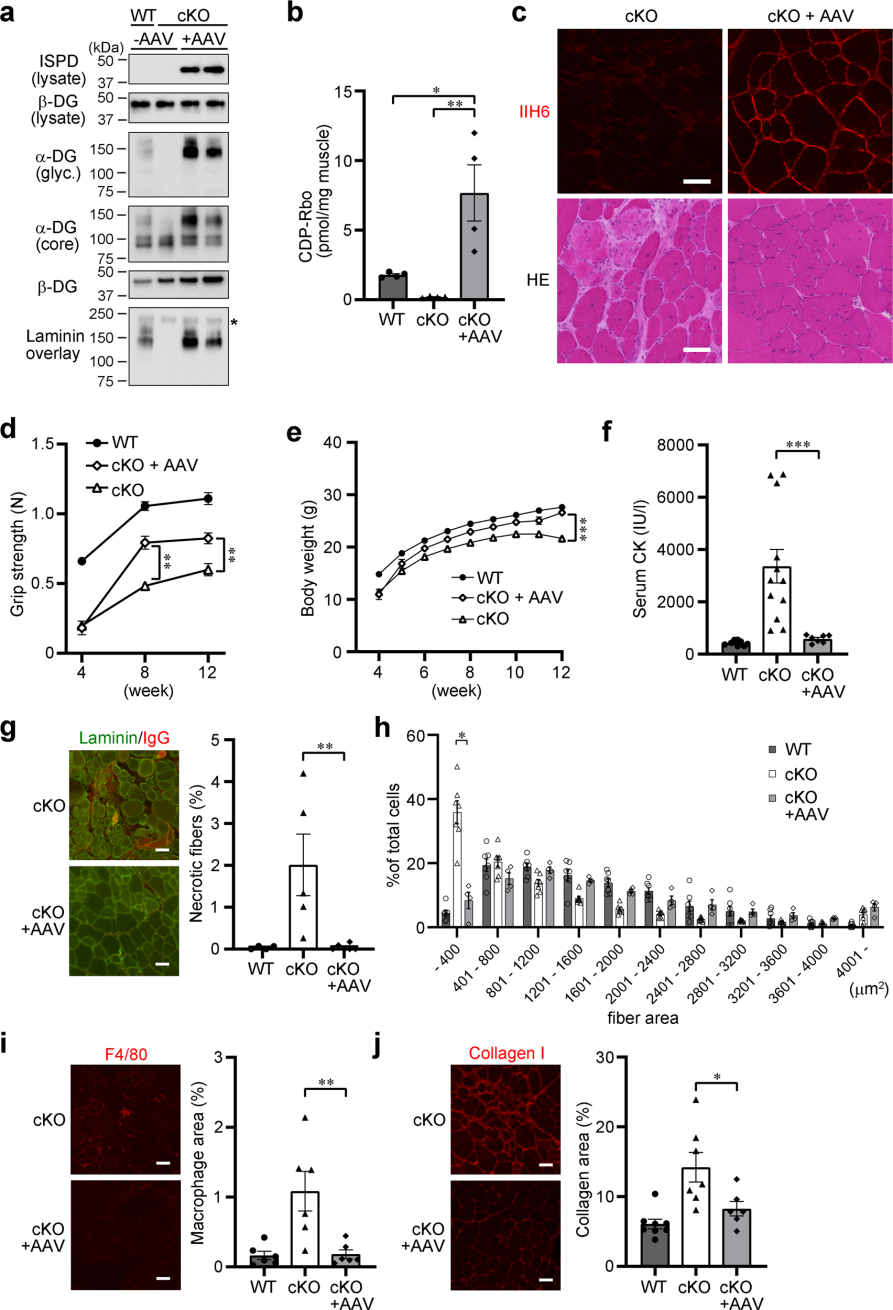
Therapeutic effects of gene replacement on the skeletal muscle pathology of Myf5-*Ispd*-cKO mice. Four-week-old Myf5-*Ispd*-cKO mice were injected with AAV9-MCK-h*ISPD* (2 × 10^12 ^v.g.) via the tail vein. After eight weeks, the skeletal muscles (TA) were analyzed and compared with non-treated Myf5-*Ispd*-cKO mice. **a** ISPD expression and α-DG glycosylation measured using western blotting and laminin overlay assays. β-DG was used as a loading control. *, endogenous laminin. **b** CDP-Rbo levels in skeletal muscle tissues (TA) after gene transfer. Data were analyzed using ANOVA with Tukey’s post hoc test (*p* = 0.015, WT vs. cKO + AAV; *p* = 0.004, cKO vs. cKO + AAV; *n* = 4). **c** Representative IIH6-immunofluorescence and HE staining images after gene transfer. **d**, **e** Temporal changes in grip strength (**d**; *p* = 0.0012 at 8 weeks*, p* = 0.0076 at 12 weeks) and body weight (**e**; *p* < 0.0001) (cKO, *n* = 39; cKO + AAV, *n* = 7). **f** Serum creatinine CK activity (*p* < 0.0001; cKO, *n* = 12; cKO + AAV, *n* = 7). **g** Immunofluorescence and quantitative analysis of necrotic fibers (*p* = 0.0043; cKO, *n* = 5; cKO + AAV, *n* = 6). **h** Quantitative analysis of myofiber size (*p* = 0.0061; cKO, *n* = 7; cKO + AAV, *n* = 4). **i**, **j** Immunofluorescence and quantitative analysis of macrophage infiltration (**i**; *p* = 0.0087; *n* = 6) and connective tissue area (**j**; *p* = 0.022; cKO, *n* = 7; cKO + AAV, *n* = 6). All data represent the mean ± SEM. Data were analyzed using the Mann-Whitney *U* test to compare cKO and cKO + AAV. *p < 0.05, **p < 0.01, and ***p < 0.001. Scale bars, 50 μm. TA, tibialis anterior.

To determine whether the dystrophic phenotype observed in Myf5-*Ispd*-cKO mice was treatable, we conducted gene replacement experiments. Previously, we demonstrated that the physical fragility of myofiber plasma membranes is a direct cause of disease onset and that adeno-associated virus (AAV) vector-mediated myofiber-selective gene expression under the control of the MCK promoter was able to ameliorate the dystrophic pathology of Myf5-*fukutin*-cKO mice^23^. Here, we introduced the *ISPD* gene into mouse skeletal muscle by intravenously administering 4-week-old Myf5-*Ispd*-cKO mice with an AAV9 vector containing the human *ISPD* cDNA under the MCK promoter (AAV9-MCK-h*ISPD)*. Therapeutic effects were examined after 2 months (12 weeks of age). Western blot analysis revealed no ISPD protein signals in non-injected WT or cKO skeletal muscle (Fig. 3a), indicating that endogenous ISPD protein levels were below detectable levels in skeletal muscle. Conversely, the skeletal muscles of cKO mice treated with AAV9-MCK-h*ISPD* displayed ISPD protein expression and recovered α-DG glycosylation (Fig. 3a). In addition, CDP-Rbo levels were dramatically higher in the AAV9-MCK-h*ISPD*-treated skeletal muscles than in non-treated Myf5-*Ispd*-cKO muscle and several times higher than in non-treated WT skeletal muscle (Fig. 3b).

Immunofluorescence analysis with IIH6 antibodies confirmed the recovery of α-DG glycosylation, while HE staining indicated the amelioration of dystrophic pathologies (Fig. 3c). Consistently, the weight of skeletal muscle was also recovered after gene replacement (Supplementary Fig. 3), while body weight and grip strength improved significantly after a single injection of AAV9-MCK-h*ISPD* (Fig. 3d, e). Serum CK levels in the treated mice also decreased to the same levels as in the WT mice (Fig. 3f) and the proportion of necrotic fibers, fiber size variation, and macrophage and connective tissue infiltration were significantly ameliorated (Fig. 3g-j). Together, these findings demonstrate that the muscular dystrophy pathology caused by *ISPD* gene loss is treatable even after disease onset.

### CDP-Rbo prodrug development

**Fig. 4:**
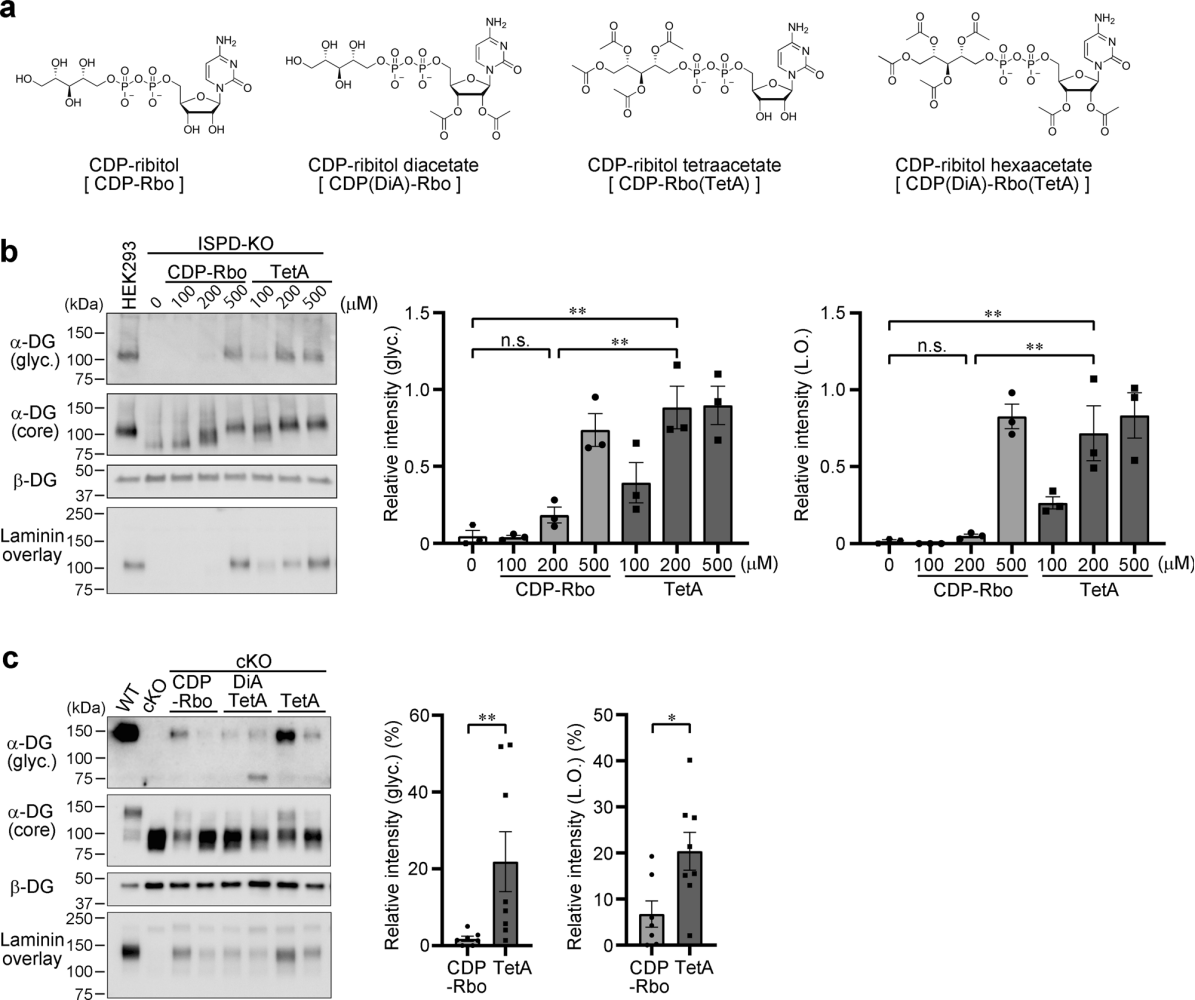
Prodrug activity of CDP-Rbo derivatives. **a** CDP-Rbo derivative structure formulae. **b** In vitro prodrug activity of CDP-Rbo derivatives in ISPD-deficient HEK293 cells. α-DG glycosylation was measured using western blot analysis with antibodies against matriglycan (glyc., IIH6) and core protein (3D7) and laminin overlay assay. β-DG was used as a loading control. DG from wild-type HEK293 cells was used as a positive control. Right: relative matriglycan signal intensity (ratio of glyc./β-DG) and laminin-binding activity (ratio of laminin binding/β-DG) compared to those in WT HEK293 cells. Data were analyzed using ANOVA with Tukey’s post hoc test. **p < 0.01 (200 μM CDP-Rbo vs. 200 μM TetA, *p* = 0.003 and 0.009; and 0 μM CDP-Rbo vs. 200 μM TetA, *p* = 0.001 and 0.007 for IIH6 and laminin overlay, respectively; *n* = 3). **c** In vivo prodrug activity of CDP-Rbo derivatives injected into skeletal muscles. CDP-Rbo or CDP-Rbo derivatives were injected twice every 3 days (days 1 and 4). Three days after the second injection (day 7), the injected muscles were harvested. α-DG glycosylation was measured using western blot analysis with antibodies against matriglycan (glyc., IIH6) and core protein (3D7) and laminin overlay assay. β-DG was used as a loading control. Right: relative matriglycan signal intensity (ratio of glyc./β-DG) and laminin-binding activity (ratio of laminin binding/β-DG) compared to those in WT muscle. Data were analyzed using the Mann-Whitney *U* test (*p* = 0.006 and 0.04 for IIH6 and laminin overlay, respectively; CDP-Rbo, *n* = 7; TetA, *n* = 8). *p < 0.05, **p < 0.01. TetA, CDP-Rbo(TetA). DiATetA, CDP(DiA)-Rbo(TetA). L.O., laminin overlay.

Since the levels of CDP-Rbo in the skeletal muscle of Myf5-*Ispd*-cKO mice increased when the *ISPD* gene was virally introduced, we hypothesized that CDP-Rbo supplementation could be an effective therapeutic strategy. Indeed, a previous study found that CDP-Rbo supplementation in *ISPD*-deficient HEK293 or HAP1 cells restored α-DG glycosylation^11^. However, CDP-Rbo displays weak membrane permeability, likely due to the presence of phosphate and hydroxy groups (Fig. 4a), which must be improved for therapeutic application. Therefore, we tried to increase the membrane permeability of CDP-Rbo by chemically modifying its hydrophilic groups with ester bonds, which can be removed by intracellular esterases after delivery. We engineered 10 different “prodrug” derivatives of CDP-Rbo (Fig. 4a and Supplementary Fig. 4), 8 of which were synthesized by replacing the hydroxy groups with acylesters (acetyl, butyl, isobutyl, and *O*-methoxycarbonyl groups). The other two types were synthesized by introducing a pentanoyloxybenzyl group on each phosphate group^28^ and six acetyl groups on the hydroxy residues. To represent the regio-chemistry of CDP-Rbo derivatives, we use simplified abbreviations here; for example, CDP(DiA)-Rbo represents the diacetyl modification on the ribose moiety of CDP and CDP-Rbo(TetA) represents the tetraacetyl modification on the ribitol moiety (Table 1 and Supplementary Fig. 4). The activities of these CDP-Rbo derivatives were evaluated by testing the restoration of α-DG glycosylation in *ISPD*-deficient HEK293 cells.

**Table 1:** Summary of prodrugs and their activity.

CDP-Rbo derivatives (simplified abbreviation)	Effective concentration* (μM)
CDP-Rbo	500
CDP(DiA)-Rbo	N.D.
CDP-Rbo(TetA)	200
CDP(DiA)-Rbo(TetA)	N.D.
CDP-Rbo(TetB)	100
CDP(DiA)-Rbo(TetB)	100–200
CDP(DiB)-Rbo(TetB)	50–100
CDP(DiA)-Rbo(TetMOC)	N.D.
CDP(DiA)-Rbo(TetIB)	200
CDP(DiA)-Rbo(TetA)-PB1	10–20
CDP(DiA)-Rbo(TetA)-PB2	50**–**200

Nomenclature of CDP-Rbo derivatives and their activity are summarized. Details for their structural formula are shown in Supplementary Fig. 4. *Effective concentration: concentration required for glycosylation recovery with levels similar to those in WT cells for CDP-Rbo(TetA) or in ISPD-KO cells treated with 200 μM CDP-Rbo(TetA) for other derivatives. N.D.; Not detected.

As expected, *ISPD*-deficient HEK293 cells displayed abnormal α-DG glycosylation, as indicated by the loss of IIH6-reactivity and reduced molecular size (Fig. 4b, lane 2), yet α-DG glycosylation was restored by supplementation with 500 μM CDP-Rbo (Fig. 4b, lane 5) or 200 μM CDP-Rbo tetraacetate [CDP-Rbo(TetA)] (Fig. 4b, lane 7). A similar pattern was observed for laminin-binding activity (Fig. 4b). These data indicate that increased IIH6-reactivity is correlated with laminin-binding activity after CDP-Rbo(TetA) treatment. Since introducing four acetyl groups to the hydroxy groups of CDP-Rbo appears to improve prodrug activity, we used 200 μM CDP-Rbo(TetA) treatment as the standard to evaluate the prodrug activities of other derivatives. While CDP-Rbo diacetate [CDP(DiA)-Rbo] and CDP-Rbo hexaacetate [CDP(DiA)-Rbo(TetA)] did not restore α-DG glycosylation at 200 μM (Supplementary Fig. 4a), derivatives containing butyl groups restored α-DG glycosylation at lower concentrations: 100 μM for CDP-Rbo tetrabutylate [CDP-Rbo(TetB)], 100 μM for CDP-Rbo tetrabutylatediacetate [CDP(DiA)-Rbo(TetB)], and 50 μM for CDP-Rbo hexabutylate [CDP(DiB)-Rbo(TetB)] (Supplementary Fig. 4b). Isobutylated CDP-Rbo [CDP(DiA)-Rbo(TetIB)] restored α-DG glycosylation at a concentration of 200 μM, but *O*-methoxycarbonylated CDP-Rbo [CDP(DiA)-Rbo(TetMOC)] could not (Supplementary Fig. 4c). Derivatives with pentanoyloxybenzyl groups [CDP(DiA)-Rbo(TetA)-PB1 and CDP(DiA)-Rbo(TetA)-PB2] restored α-DG glycosylation at concentrations below 50 μM, indicating that they had the greatest prodrug activity in vitro (Supplementary Fig. 4d).

To examine whether the CDP-Rbo derivatives were also able to restore α-DG glycosylation in vivo, we injected the skeletal muscle of Myf5-*Ispd*-cKO mice with 8 derivatives [CDP-Rbo(TetA), CDP(DiA)-Rbo(TetA), CDP-Rbo(TetB), CDP(DiA)-Rbo(TetB), CDP(DiA)-Rbo(TetIB), CDP(DiB)-Rbo(TetB), CDP(DiA)-Rbo(TetA)-PB1, and CDP(DiA)-Rbo(TetA)-PB2] twice a week, and the skeletal muscles were harvested 3 days after the second injection. As glycosylation was recovered 24 h after the addition of CDP-Rbo derivatives in vitro and the recovered α-DG glycosylation needs to remain in vivo for a certain period of time, we selected the 3-day post-injection point for the initial in vivo prodrug screening. Although weak IIH6 signals were sometimes detected after treatment with unmodified CDP-Rbo or CDP(DiA)-Rbo(TetA), no obvious restoration in α-DG molecular size was observed (Fig. 4c, lanes 3–6). However, treatment with CDP-Rbo(TetA) restored the IIH6-positivity, normal molecular size, and laminin-binding activity of α-DG more efficiently (Fig. 4c, lanes 7 and 8). The quantitative evaluation revealed that α-DG glycosylation improved significantly after CDP-Rbo(TetA) treatment compared to CDP-Rbo treatment (Fig. 4c, right graph). Other derivatives failed to restore α-DG glycosylation (Fig. 4c and Supplementary Fig. 5a and 5b). Treatments with CDP(DiB)-Rbo(TetB), CDP(DiA)-Rbo(TetA)-PB1, or CDP(DiA)-Rbo(TetA)-PB2 resulted in edema or severe muscle atrophy, indicating that these derivatives were toxic in vivo (Supplementary Fig. 5c). Therefore, we decided to test the therapeutic activity of CDP-Rbo(TetA).

### Therapeutic effects of CDP-Rbo prodrug, CDP-Rbo(TetA)

**Fig. 5:**
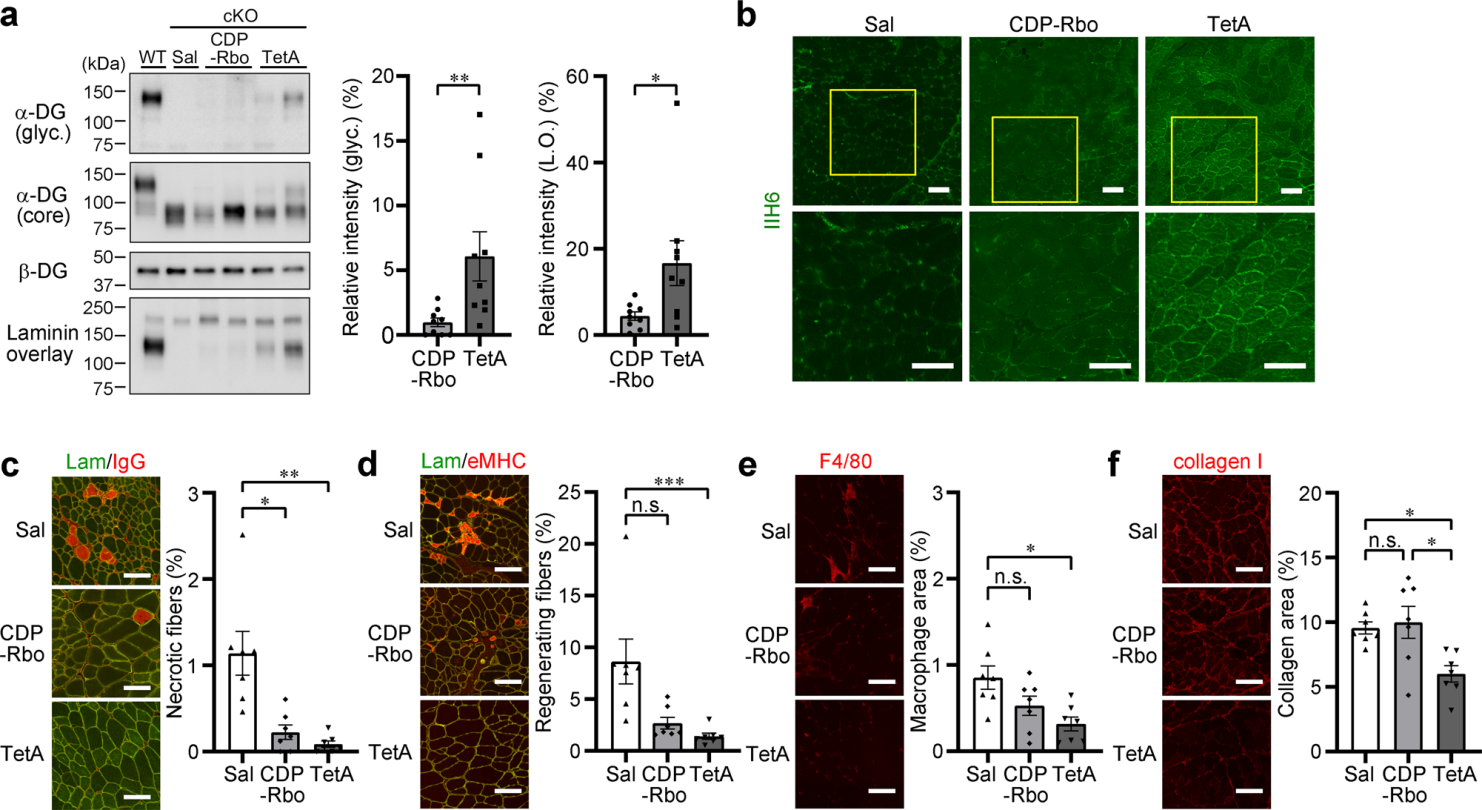
CDP-Rbo prodrug administration ameliorates the dystrophic pathology of Myf5-*Ispd*-cKO mice. The TA muscle of 4-week-old mice was injected with CDP-Rbo or CDP-Rbo(TetA) twice weekly (six times in total). Ten days after the final injection, muscles were harvested and subjected to biochemical and histopathological analyses. Saline-treated muscles were used as controls (Sal). **a** α-DG glycosylation was measured using western blot and laminin overlay analyses after CDP-Rbo or CDP-Rbo(TetA) administration. Right: relative matriglycan signal intensity (ratio of glyc./β-DG) and laminin-binding activity (ratio of laminin binding/β-DG) compared to those in WT muscle. Data were analyzed using the Mann-Whitney *U* test (*p* = 0.0039 and 0.024 for IIH6 and laminin overlay respectively, *n* = 9). **b** IIH6-immunofluorescence analysis after CDP-Rbo or CDP-Rbo(TetA) administration. Areas indicated by yellow squares are enlarged in the lower panels. The images are representative of at least seven mice analyzed in each group. **c**–**f** Immunofluorescence and quantitative analysis of necrotic fibers (**c**; *p* = 0.019, CDP-Rbo vs. Sal, *p* = 0.001, CDP-Rbo(TetA) vs. Sal), regenerating fibers (**d**; *p* = 0.0005, CDP-Rbo(TetA) vs. Sal), macrophage infiltration (**e**; *p* = 0.02, CDP-Rbo(TetA) vs. Sal), and connective tissue area (**f**; *p* = 0.037, CDP-Rbo(TetA) vs. Sal, *p* = 0.023, CDP-Rbo(TetA) vs. CDP-Rbo). *n* = 7 per group. Data represent the mean ± SEM. Data were analyzed using the Kruskal-Wallis ANOVA test followed by Dunn’s multiple comparisons. *p < 0.05, **p < 0.01, and ***p < 0.001. Scale bars, 100 μm. TA, tibialis anterior. TetA, CDP-Rbo(TetA). L.O., laminin overlay.

To examine whether CDP-Rbo(TetA) has therapeutic benefits, we injected the lower leg muscles (TA and calf) of 4-week-old Myf5-*Ispd*-cKO mice with CDP-Rbo or CDP-Rbo(TetA) for 3 weeks (two injections/week; Supplementary Fig. 6a). Skeletal muscle samples were prepared at 8 weeks of age, 10 days after the last injection. We considered that 10 days would be sufficient time to recover from any possible experimental damage, such as needle wounds, and to test the true effects of the prodrug treatment. Western blot analysis revealed that α-DG glycosylation was restored in some individuals treated with either CDP-Rbo or CDP-Rbo(TetA) (Fig. 5a). Although the relative α-DG glycosylation signals from the treated muscles were less than 10 % of those observed in WT muscles, CDP-Rbo(TetA) treatment significantly improved α-DG glycosylation and laminin-binding compared to CDP-Rbo (Fig. 5a, right). Moreover, immunofluorescence staining confirmed the partial restoration of IIH6-positive fibers in CDP-Rbo(TetA)-injected My5-*Ispd*-cKO mice (Fig. 5b). Consistently, HE staining suggested that CDP-Rbo(TetA) treatment ameliorated the dystrophic pathology (Supplementary Fig. 6b). Quantitative histopathological analyses showed that CDP-Rbo(TetA)- or CDP-Rbo-treated Myf5-*Ispd*-cKO muscles contained a significantly lower proportion of necrotic fibers (Fig. 5c) and smaller fibers (Supplementary Fig. 6c), with CDP-Rbo(TetA) showing lower P values. CDP-Rbo(TetA) treatment, but not CDP-Rbo treatment, significantly reduced the proportion of regenerating fibers and macrophage infiltration compared to the case for the non-treated Myf5-*Ispd*-cKO mice (Fig. 5d, e). Furthermore, CDP-Rbo(TetA) treatment significantly reduced the degree of fibrosis, compared to the CDP-Rbo treatment (Fig. 5f). These data indicate that CDP-Rbo(TetA) exerts more obvious therapeutic effects than CDP-Rbo.

Having demonstrated the therapeutic efficacy of the two treatments, we examined the stability of CDP-Rbo and CDP-Rbo(TetA) in plasma and skeletal muscle. Both CDP-Rbo and CDP-Rbo(TetA) were degraded at similar rates in mouse plasma, with approximately 10 % remaining intact after incubation for 60 min (Supplementary Fig. 6d). However, both CDP-Rbo and CDP-Rbo(TetA) degraded at a slower rate in human plasma, with around 40 % remaining after incubation for 60 min (Supplementary Fig. 6d). To confirm the conversion from CDP-Rbo(TetA) to CDP-Rbo as well as the stability of CDP-Rbo in mouse skeletal muscle, we extracted CDP-Rbo from skeletal muscle 72 h after a single injection of either CDP-Rbo or CDP-Rbo(TetA). Quantitative LC/MS analysis detected high CDP-Rbo levels in CDP-Rbo(TetA)-injected mice (Supplementary Fig. 6e), indicating that CDP-Rbo(TetA) was efficiently converted into CDP-Rbo that remained in skeletal muscle for at least 72 h.

We tested systemic treatment with CDP-Rbo(TetA) via tail vein injection. The amount of CDP-Rbo in the skeletal muscle was not significantly increased in the CDP-Rbo(TetA)-treated mice compared to that in non-treated Myf5-*Ispd*-cKO mice (Supplementary Fig. 7a). Although we did not see signs of increased α-DG glycosylation and laminin-binding activity in TA muscles of CDP-Rbo(TetA)-treated Myf5-*Ispd*-cKO mice (Supplementary Fig. 7b), interestingly, quadriceps from some mice showed minor increases in α-DG glycosylation and laminin binding after CDP-Rbo(TetA)-treatment, and such increases were not observed in CDP-Rbo-treated Myf5-*Ispd*-cKO mice (Supplementary Fig. 7c). These observations can be attributed to the differences in the stability of CDP-Rbo and glycosylated α-DG in the tissue and/or tissue-dependent delivery efficacy of CDP-Rbo(TetA). These data suggest that the prodrug activity of CDP-Rbo(TetA) could be potentially observed when it is systemically administered; however, there is room for improving its stability and delivery efficiency to test long-term treatment.

We also tested the systemic administration of ribitol to Myf5-*Ispd*-cKO mice. Ribitol supplementation to normal mouse or cells has been known to increase the production of CDP-Rbo, possibly through increasing ribitol-5-phosphate, a substrate for ISPD^17^. Ribitol administration via drinking water dramatically increased the levels of CDP-Rbo in WT mice; however, this effect was not observed in Myf5-*Ispd*-cKO mice (Supplementary Fig. 8a). Minor increases in CDP-Rbo in Myf5-*Ispd*-cKO mice after ribitol treatment could be derived from non-muscle cells that were not targeted by Myf5-Cre recombination. Accordingly, we observed little effect on glycosylation recovery in Myf5-*Ispd*-cKO mice after the systemic ribitol administration (Supplementary Fig. 8b); further, grip strength, body weight, muscle weight, and histopathology were not improved (Supplementary Fig. 8c–g). We also tested intramuscular injections of ribitol in excess amount into the TA and calf muscles of Myf5-*Ispd*-cKO mice. In most of the treated mice, CDP-Rbo levels and α-DG glycosylation failed to increase; however, a minor increment in glycosylation signals was observed in one mouse (Supplementary Fig. 8h and i). This could be due to the minimally-resided Ispd activity because of the cKO system. Together, these data show that ISPD is a protein responsible for producing CDP-Rbo upon ribitol treatment and that ribitol supplementation therapy depends on the ISPD activity.

**Fig. 6:**
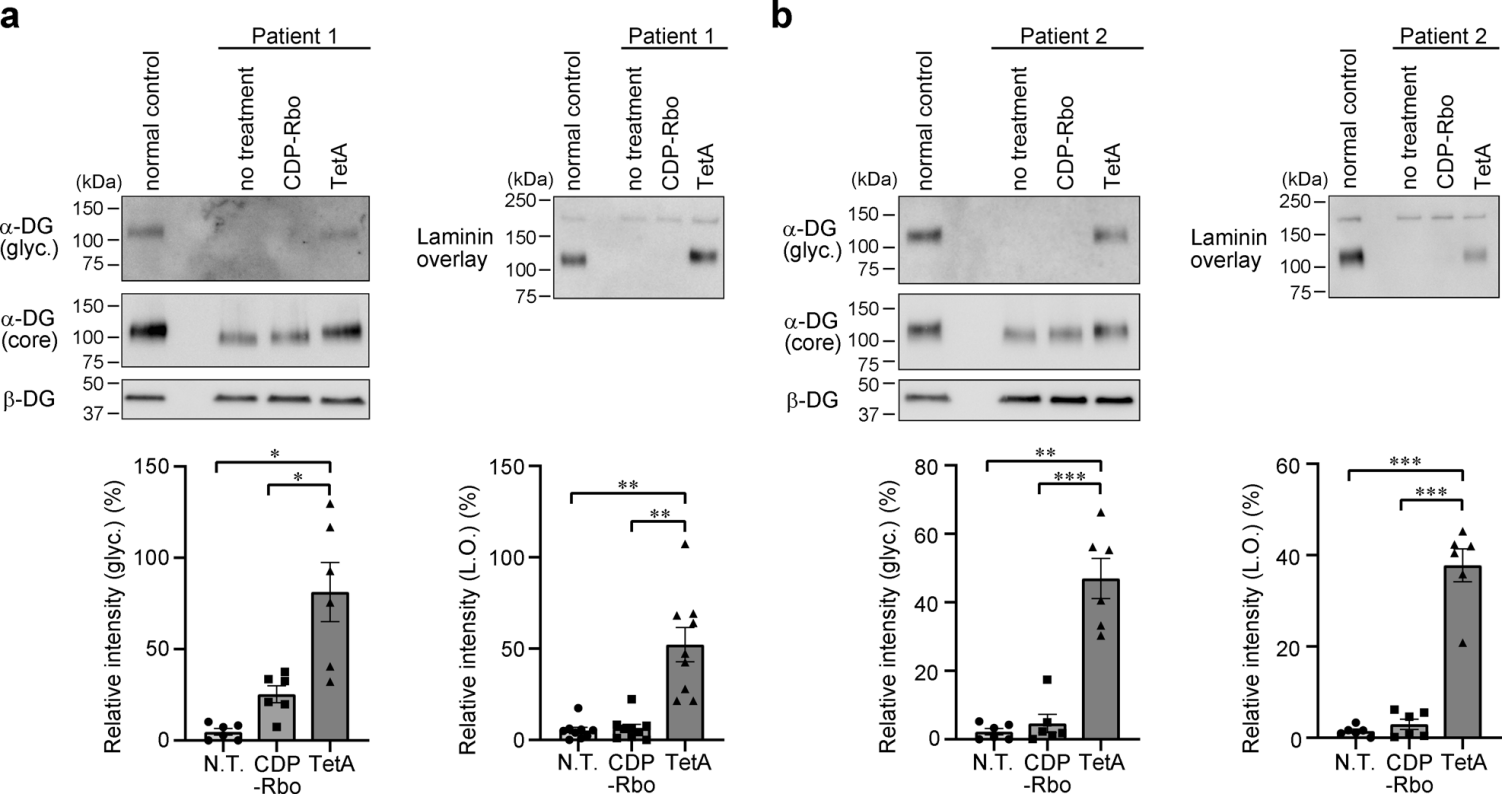
CDP-Rbo(TetA) treatment recovers α-DG glycosylation in patient fibroblasts. Two different fibroblast cell lines derived from human patients (**a**; Walker-Warburg syndrome patient, **b**; muscle-eye-brain disease patient) with ISPD-deficiency were treated with CDP-Rbo (200 μM) or CDP-Rbo(TetA) (200 μM) for 24 h. α-DG glycosylation was measured using western blot analysis with antibodies against matriglycan (glyc., IIH6) and core protein (3D7) or a laminin overlay assay. β-DG was used as a loading control. DG from normal human fibroblasts was used as a positive control. Bottom: relative matriglycan signal intensity (ratio of glyc./β-DG) and laminin-binding activity (ratio of laminin binding/β-DG) compared to those in normal human dermal fibroblasts (NHDF). All data represent the mean ± SEM. Data were analyzed using Brown–Forsythe and Welch ANOVA test followed by Dunnett’s T3 multiple comparisons (IIH6 of patient 1, *p* = 0.014, N.T. vs. TetA; *p* = 0.042, CDP-Rbo vs. TetA, *n* = 6; laminin overlay of patient 1, *p* = 0.002, N.T. vs. TetA; *p* = 0.003, CDP-Rbo vs. TetA, *n* = 9; IIH6 of patient 2, *p* = 0.002, N.T. vs. TetA; *p* = 0.0009, CDP-Rbo vs. TetA, *n* = 6; and laminin overlay of patient 2, *p* = 0.0005, N.T. vs. TetA; *p* = 0.0003, CDP-Rbo vs. TetA, *n* = 6). *p < 0.05, **p < 0.01, and ***p < 0.001. TetA, CDP-Rbo(TetA). N.T., no treatment. L.O., laminin overlay.

Finally, we treated human fibroblasts derived from two different ISPD-deficient patients with CDP-Rbo or CDP-Rbo(TetA) to confirm the efficacy of these treatments (Fig. 6). Prior to treatment, the cells displayed abnormal α-DG glycosylation, as indicated by the loss of IIH6-immunoreactivity and reduced molecular size compared to normal fibroblasts. CDP-Rbo(TetA) increased the molecular size of α-DG, produced IIH6-immunoreactivity, and recovered the laminin-binding activity of α-DG (Fig. 6, lane 4); however, CDP-Rbo treatment was unable to achieve these effects. Quantification of IIH6-reactivity and laminin-binding activity of α-DG confirmed prodrug activity of CDP-Rbo(TetA) in human cells (Fig. 6). Together, these results indicate that CDP-Rbo prodrug supplementation in skeletal muscles exerts therapeutic benefits in *ISPD*-deficient muscular dystrophy.

## Discussion

In this study, we generated skeletal muscle-selective Myf5-*Ispd*-cKO mice and confirmed that ISPD is responsible for CDP-Rbo production in vivo and that the loss of CDP-Rbo production leads to muscular dystrophy. We also demonstrated that AAV vector-mediated gene replacement effectively improves muscular dystrophy caused by ISPD deficiency. Moreover, we developed an original series of CDP-Rbo derivative prodrugs, one of which was able to rescue α-DG glycosylation and ameliorate muscular dystrophic changes in vivo.

*ISPD* gene mutations have been shown to cause Walker-Warburg syndrome and the gene is predicted to encode a key enzyme in DG *O*-mannosylation^14^. In a zebrafish study, *ISPD* ortholog knockdown disrupted myotendinous junctions and muscle degeneration^14^, suggesting that ISPD may play important roles in skeletal muscle via α-DG glycosylation. Later studies showed that *ISPD* encodes an enzyme that synthesizes CDP-Rbo from Rbo5P and CTP;^11,16,17^ however, no studies have yet determined whether ISPD is responsible for CDP-Rbo production and α-DG glycosylation in skeletal muscle in vivo. Our data, including gene rescue experiments, clearly demonstrated that ISPD is responsible for producing CDP-Rbo in mammalian skeletal muscle as well as α-DG glycosylation. Consistent with the recessive nature of *ISPD*-deficient muscular dystrophy, heterozygous mice did not display any pathological abnormalities in skeletal muscle or α-DG glycosylation; however, CDP-Rbo levels in the Het mice were almost half of those in WT mice. Myf5-*Ispd*-cKO muscle contains CDP-Rbo at a concentration of ~0.3 pmol/mg tissue and shows almost complete defects in α-DG glycosylation, suggesting that at least sub pmol/mg tissue CDP-Rbo is required for maintaining α-DG glycosylation and muscle health. Thus, administering skeletal muscle with small amounts of CDP-Rbo may exert therapeutic efficacy in *ISPD*-deficient muscular dystrophy.

The Myf5-*Ispd*-cKO mice exhibited severe pathological hallmarks including fiber size variation and fibrotic tissue infiltration, which are often observed during the clinical course of congenital muscular dystrophy caused by ISPD mutations^18^. When 4-week-old Myf5-*Ispd*-cKO mice with a dystrophic pathology were administered the AAV9-MCK-h*ISPD* vectors, we observed a dramatic improvement in muscle pathology without obvious adverse effects, indicating that therapeutic effects can even be achieved after disease onset. Recent gene therapies for genetic neuromuscular disorders have been rapidly developed for clinical applications;^29,30^ therefore, the data provided by our study could accelerate the development of AAV-mediated *ISPD* gene therapy. Moreover, this therapy could be used for both Fukutin- and FKRP-deficient cases, since ISPD overexpression increased CDP-Rbo levels in skeletal muscle, which may boost residual RboP-transferase activity in disease-causing Fukutin or FKRP mutant proteins to restore α-DG glycosylation. Consistently, Cataldi *et al*. reported that increasing CDP-Rbo levels via ribitol supplementation and ISPD overexpression can restore α-DG glycosylation and exert therapeutic effects in FKRP^P448L^ mutant mice^31,32^.

Although gene replacement is a feasible therapeutic strategy for ISPD-deficient and related muscular dystrophies, there are several issues regarding clinical application. For instance, AAV vector administration can lead to side effects such as immune responses and liver failure, particularly during high-dose therapy^33^. In addition, the presence of neutralizing antibodies against viruses poses a critical problem for AAV-mediated gene therapies targeting adult neuromuscular disease, while the extremely high cost of treatment can be a major hurdle for patients. To overcome these potential issues, alternative therapeutic options are also desirable; therefore, we generated a series of CDP-Rbo prodrugs to investigate whether supplementation with CDP-Rbo, the enzymatic product of ISPD, could effectively treat ISPD-deficiency. The administration of Myf5-*Ispd*-cKO skeletal muscle with CDP-Rbo(TetA) increased CDP-Rbo levels and restored α-DG glycosylation, with long-term CDP-Rbo(TetA) treatment displaying therapeutic effects without obvious toxicity. Therefore, supplementation with CDP-Rbo prodrugs could be an effective treatment for ISPD-deficient muscular dystrophy. Regarding the extent to which glycosylation should be recovered for obtaining therapeutic benefits, Myf5-*Ispd*-cKO mice with mild phenotype showed less than 10 % glycosylation compared with WT mice. This suggests that at least 5–10 % glycosylation can lead to beneficial effects (Supplementary Fig. 2c). Accordingly, a previous report also suggested that only partial restoration of glycosylation was sufficient to prevent muscular dystrophy in another dystroglycanopathy model mouse^34^. Our data showed ~20 % and ~6 % of glycosylation recovery at 3 days and 10 days, respectively, after CDP-Rbo(TetA) intramuscular injections; however, since the recovery of glycosylation may be limited locally and CDP-Rbo(TetA) exhaustion can lead to the turnover of glycans, these values could be underestimated. Of course, more widespread delivery methods will likely improve the therapeutic benefits, and should, therefore, be developed in the future. It would also be necessary to investigate the relationship between glycosylation recovery and muscle physiological function for future therapeutic approaches.

Although we found that CDP-Rbo administration exerted some therapeutic effects, this treatment was significantly less effective than CDP-Rbo(TetA), for which there are several possible explanations. Our in vitro studies confirmed that treatment with high CDP-Rbo concentrations led to some membrane permeability, while dystrophic myofibers may show increased membrane leakiness for low-molecular-weight compounds. However, the increased therapeutic efficacy of CDP-Rbo(TetA) compared to CDP-Rbo may be attributed to the higher membrane permeability of CDP-Rbo(TetA), since the stability of CDP-Rbo(TetA) and CDP-Rbo in plasma did not differ. As for the therapeutic benefit of CDP-Rbo(TetA), the reduction in necrotic fibers observed in CDP-Rbo(TetA)-treated muscle is likely to result from restored α-DG glycosylation, as DG physically protects myofibers from mechanical damage in a glycosylation-dependent manner^23,35^. Therefore, it is reasonable to propose that fibrotic tissue and immune cell infiltration were significantly suppressed in the CDP-Rbo(TetA)-treated muscle. Although α-DG glycosylation was not restored in some mice treated with CDP-Rbo(TetA), therapeutic effects were still observed. This may be due to insufficient CDP-Rbo(TetA) delivery, individual differences in responsiveness to CDP-Rbo(TetA), or particularly α-DG glycan turnover in the skeletal muscle tissues dissected 10 days after the final CDP-Rbo(TetA) administration. Consistently, previous studies have reported a time lag between the loss of glycosylation and disease onset in mouse models^23,24^. Alternatively, it is possible that ISPD and/or CDP-Rbo has targets other than α-DG, which may be involved in the beneficial effects of ameliorating the dystrophic pathology. In addition, there is a possibility that CDP-Rbo per se has some therapeutic effects, such as anti-inflammatory action. These possibilities should be investigated in the future.

Conversely, CDP-Rbo(TetA) administration may not have achieved dramatic therapeutic effects due to the limited spatial expansion of CDP-Rbo(TetA) following local injections or the dissociation of acetyl groups from CDP-Rbo(TetA) outside of myofibers due to extracellular esterases resulting in the loss of membrane permeability. The latter hypothesis may be supported by the fact that CDP-Rbo levels in the skeletal muscles 72 h after CDP-Rbo(TetA) injection (Supplementary Fig. 6e) were higher than those in WT skeletal muscles under physiological conditions (Fig. 1B). In addition, CDP-Rbo and CDP-Rbo(TetA) degradation rates did not differ in blood plasma, suggesting that acetyl groups do not protect CDP-Rbo structural and chemical stability. Although the mechanisms underlying degradation in blood plasma remain unknown, it is possible that the phosphodiester-linkage in CDP-Rbo may be a target of nonselective phosphodiesterase in the blood. Therefore, further studies are required to improve prodrug activity by modifying CDP-Rbo with more functional groups on hydroxy- or phosphate-groups to allow more widespread delivery and/or protect against degradation. For example, the application of ProTide (prodrugs of nucleotides) technology, which has been utilized for the development of remdesivir^36^, may be a potential method to improve prodrug activity. In addition, the efficacy of CDP-Rbo supplementation therapy could be improved by combination with a drug delivery system (DDS).

Previous studies have found that ribitol supplementation can restore α-DG glycosylation in ISPD-deficient fibroblasts^17^ and thus could represent a novel therapeutic strategy for dystroglycanopathy. Exogenous ribitol likely penetrates the cell membrane and is converted to ribitol 5-phosphate, an ISPD substrate, via an unidentified intracellular metabolic pathway. Consequently, increased intracellular ribitol 5-phosphate levels may enhance the rate of CDP-Rbo production by disease-causing ISPD mutant proteins. Such “boost” effects can be observed if missense mutations are present near the substrate recognition domain of ISPD; however, ribitol supplementation may not be applicable to all missense or nonsense mutations. Consistently, a recent study reported that ribitol administration restores CDP-Rbo levels and α-DG glycosylation in a mutation-dependent manner^37^. In addition, increased levels of primary or intermediate metabolites during enzyme chain reactions may activate unexpected pathways and cause detrimental effects, meaning that our CDP-Rbo supplementation strategy could be applied to any type of ISPD mutation. Interestingly, Cataldi *et al*. proposed that ribitol supplementation restores α-DG glycosylation in FKRP^P448L^ mutant mice and ameliorates their dystrophic pathology^31^. Although the detailed mechanism underlying the therapeutic effects of ribitol supplementation in FKRP-deficient cases remains unclear, CDP-Rbo supplementation therapy could also be applicable to Fukutin- or FKRP-deficient muscular dystrophies if increased CDP-Rbo levels could enhance the residual RboP-transferase activities of Fukutin or FKRP mutant proteins. Therefore, we propose that CDP-Rbo supplementation is a rational strategy for treating certain types of muscular dystrophy with RboP-modification defects.

Our data also provide important insights into the treatment of other glycosylation-related diseases and basic studies of glycobiology and cell biology. Some congenital disorders of glycosylation (CDGs) are caused by abnormal sugar nucleotide metabolism, such as defects in GDP-Man (*PMM2*-CDG) biosynthesis or an unbalanced UDP-Gal/UDP-GlcNAc ratio (*PGM1*-CDG). *SLC35C1*-CDG is caused by mutations in *SLC35C1*, which encodes a GDP-fucose transporter in the Golgi apparatus, and it has been proposed that elevated GDP-fucose levels compensate for the reduced affinity of mutant SLC35C1^38^. Some congenital myasthenic syndromes (CMSs) are also caused by defects in nucleotide sugar biosynthesis pathways; therefore, our findings support the theory that nucleotide sugar supplementation could be a potential therapeutic strategy for these diseases. In addition, our screening results for the 10 CDP-Rbo derivatives generated in this study could provide important information for the development of nucleotide sugar prodrugs. For instance, our data demonstrate the structure-function relationship between acyl groups and prodrug activity, suggesting that the position and number of acyl groups on hydroxyl groups should also be considered. Phosphate groups possess a higher polarity and hydrophilicity than hydroxyl groups and therefore serve as barriers to membrane permeability. In fact, the modification of pyrophosphate groups on CDP-Rbo dramatically increased prodrug activity in vitro. However, our data also suggest that the in vivo toxicity of functional groups in derivatives should be carefully considered. In our 10 CDP-Rbo derivatives, in vivo toxicity seemed to correlate to structural complexity, such as the number of carbon atoms and branches. Considering the membrane permeability and toxicity, we concluded that CDP-Rbo(TetA) was the best prodrug among the 10 derivatives developed in this study.

Our data provide useful information for the design and development of compounds that contain sugar nucleotides or pyrophosphate groups for applications in clinical settings or as chemical biology tools. Together, our data demonstrate that ISPD-deficient muscular dystrophy can be treated via strategies such as gene replacement and CDP-Rbo supplementation. Future studies should improve the therapeutic outcomes of CDP-Rbo prodrugs and examine their combination with DDS.

## Methods

### Generation of Myf5-*Ispd-*cKO mice

Heterozygous *Ispd* flox mice (*Ispd*^*lox/+*^) were generated by the mouse biology program (MBP) at the University of California, Davis (C57BL/6N-*Ispd*^*em2Mbp*^/Mmucd; RRID: MMRRC_037583-UCD)^21^ and were intercrossed to obtain homozygous floxed mice (*Ispd*^*lox/lox*^). Myf5-Cre knock-in (KI) mice (*Myf5-Cre*^*KI*^ (+)) obtained from The Jackson Laboratory (B6.129S4-*Myf5*^*tm3(cre)Sor*^/J; Stock No 007893)^22^ were backcrossed for more than six generations with C57BL/6 mice before crossing with *Ispd*^*lox/lox*^ mice. Heterozygous *Ispd*^*lox/+*^ mice carrying Myf5-Cre [*Ispd*^*lox/+*^: *Myf5-Cre*^*KI*^ (+)] were bred with *Ispd*^*lox/lox*^ mice to obtain Myf5-*Ispd*-cKO mice. This breeding strategy produced the following three genotypes: *Ispd*^*lox/lox*^:*Myf5-Cre*^*KI*^ (-), used as WT control; *Ispd*^*lox/+*^:*Myf5-Cre*^*KI*^ (+), used as heterozygous control (Het); and *Ispd*^*lox/lox*^:*Myf5-Cre*^*KI*^ (+), used as cKO. Tail DNA was genotyped using PCR analysis with the primer sequences shown in Supplementary Table 1. PCR conditions are available on request. The mice were maintained in accordance with the ARRIVE guidelines and animal care guidelines of Kobe University and Ehime University. All animal experiments were approved by the Animal Care and Use Committees of Kobe University Graduate School of Medicine (P150605, P180901, and P200409) and Ehime University Graduate School of Medicine (05-O-70-1).

### Protein preparation and western blot analysis

DG was enriched from solubilized skeletal muscle, as described previously^39^. Briefly, mouse skeletal muscles were solubilized in Tris-buffered saline (TBS) containing 1% Triton X-100 and a protease inhibitor cocktail (Nacalai Tesque, Kyoto, Japan). The solubilized fraction (total lysate) was incubated with wheat germ agglutinin (WGA)-agarose beads (Vector Laboratories, Burlingame, CA, USA) at 4 °C for 16 h. DG was eluted using Laemmli sample buffer and proteins were separated using 4–15% linear gradient SDS-PAGE (Bio-Rad, Hercules, CA, USA). Samples were transferred to polyvinylidene fluoride membranes (Merck Millipore, Darmstadt, Germany) which were blocked with 5% skim milk in TBS containing 0.1 % Tween 20 (TBST), incubated with primary antibodies, and then incubated with horseradish peroxidase-conjugated secondary antibodies. After washing with TBST, the blots were developed using chemiluminescence (Supersignal West Pico PLUS, Thermo Fisher Scientific, Waltham, MA, USA; and ECL Prime, GE Healthcare, Chicago, IL, USA). Laminin overlay assay was performed as described previously^23^. Whole gel images are shown in Source Data. The primary and secondary antibodies used are listed in Supplementary Tables 2 and 3.

### Histological and immunofluorescence analyses

Muscles tissues were embedded in OCT compound (Sakura Finetek, Tokyo, Japan), frozen in liquid-nitrogen-cooled isopentane, and cryosectioned (7 μm-thick). HE staining and IIH6-immunofluorescence analysis were performed as described previously^40^. For immunofluorescence staining, sections were blocked with 3 % bovine serum albumin (BSA) in phosphate-buffered saline (PBS) at room temperature for 1 h and then incubated with primary antibodies diluted in 1% BSA overnight at 4 °C. For immunostaining with antibodies against macrophages (F4/80), embryonic myosin (F1.652), and matriglycan on α-DG (IIH6), the slides were blocked using an M.O.M kit (Vector Laboratories), washed with PBS, and incubated with Alexa Fluor 488-conjugated or Alexa Fluor 555-conjugated secondary antibodies (Thermo Fisher Scientific) at room temperature for 30 min. The slides were mounted for HE staining and immunofluorescence analysis using Permount (Thermo Fisher Scientific) and TISSU MOUNT (Shiraimatsu Kikai, Osaka, Japan), respectively. Images were observed using fluorescence microscopy (BZ-9000 microscope, Keyence, Osaka, Japan). The primary and secondary antibodies used are listed in Supplementary Tables 2 and 3.

### Adeno-associated viral gene transfer

To generate *the ISPD*-encoding AAV9 vector, the complete open reading frame of the human *ISPD* gene was cloned into the pAAV-IRES-hrGFP vector and the MCK promoter was subcloned from the AAV9-MCK-*fukuin* vector^23^. The recombinant *ISPD*-encoding AAV9 vector was produced as described previously^41^. Four-week-old Myf5-*Ispd*-cKO mice were injected with AAV vectors (AAV9-MCK-h*ISPD*; 2 × 10^12^ vector genome) via the tail vein. Muscle samples were prepared 8 weeks after injection.

### CDP-Rbo and derivatives

CDP-Rbo and its derivatives were custom made by the Peptide Institute (Osaka, Japan). The structural formulae, NMR charts, and HPLC charts of these compounds are shown in Supplementary Fig. 9.

### In vitro evaluation of CDP-Rbo derivative prodrug activities

HEK293 cells and human fibroblasts were cultured in high glucose Dulbecco’s modified Eagle’s medium (DMEM) with L-glutamine, phenol red, and sodium pyruvate (Wako Pure Chemical Industries, Osaka, Japan) supplemented with fetal bovine serum (10%), penicillin (100 units/mL), and streptomycin (100 μg/mL) at 37 °C in a humidified 5% CO_2_ atmosphere. Human primary fibroblasts were reported previously (patient 1, homozygous, p.Arg205His; patient 2, heterozygous, p.Ala122Pro/p.Arg268X)^14,37^. Informed consent was obtained from patients or their legal representatives for use of fibroblasts. Fibroblast cultures were collected as part of clinical care. Residual, de-identified material was used in this study for functional measurements under ethics agreements from the Radboud University Medical Center, The Netherlands (2020–6588). Patients 1 and 2 showed symptoms of Walker-Warburg syndrome and muscle-eye-brain disease, respectively^14,37^. Normal human fibroblasts were obtained from PromoCell (Heidelberg, Germany; D10052). The use of all clinical samples was approved by the Human Ethics Review Committees of Kobe University Graduate School of Medicine (1088).

*ISPD*-disrupted HEK293 cells were plated in 6-well culture dishes the day before CDP-Rbo derivatives (dissolved in water) were added, as described previously^11^. For CDP-Rbo(TetA) treatment, human fibroblasts were cultured on 100-mm dishes; 200 μM CDP-Rbo or CDP-Rbo(TetA) was added at ~80% confluency, and then the fibroblasts were further cultured for 24 h. After 24 h, the cells were solubilized with TBS containing 1% Triton X-100 and protease inhibitors (Nacalai Tesque), incubated with WGA-agarose beads at 4 °C for 16 h, and DG was eluted using Laemmli sample buffer.

### Administration of Myf5-*Ispd*-cKO mice with CDP-Rbo, its derivatives, and ribitol

To screen CDP-Rbo derivatives in vivo, TA muscle was injected with 40 μL of each derivative (100 mM in saline) twice every 3 days (days 1 and 4). At least two mice were tested per derivative. Three days after the second injection (day 7), the muscles were harvested and α-DG glycosylation was detected using western blot analysis. For long-term treatment (4 weeks), TA muscle was injected with CDP-Rbo or CDP-Rbo(TetA) (40 μL, 100 mM in saline) twice a week (six times in total). Saline-injected mice were used as controls. Ten days after the final injection, muscles were harvested for biochemical and histopathological analyses.

To test the systemic delivery of CDP-Rbo(TetA), CDP-Rbo(TetA) (100 mM in saline, 150 μL) was injected via tail vein twice a week. After 3 days of the second injection, the skeletal muscles were harvested for testing DG glycosylation and CDP-Rbo. For the ribitol treatments, 5 % ribitol was systemically administered via drinking water for 4 weeks or directly injected into TA (40 μL) and calf (60 μL) twice (on days 1 and 4). The intramuscularly injected muscles were harvested 3 days after the second injection (day 7).

### Quantitative analysis of muscle pathology

The proportion of myofibers with centrally located nuclei and the number of necrotizing (IgG-positive) and regenerating fibers (embryonic myosin-positive) were counted in at least 1,000 fibers per mouse. To evaluate connective tissue and macrophage infiltration, collagen I and F4/80 immunofluorescence signals, were quantified using ImageJ software (https://imagej.nih.gov/ij/), respectively. To assess myofiber size variation, the area of individual myofibers on transverse sections was measured using ImageJ software after laminin immunostaining. Serum creatine kinase (CK) activity was analyzed using a CPK Kit (Wako Chemical, Osaka, Japan). Grip strength was measured in 10 consecutive trials per mouse using a strength meter (Ohara Ika Sangyo, Tokyo, Japan), with the top and bottom 20 % of values excluded to obtain a mean value. At least 40 non-treated mice were analyzed per test (*n* indicated in figure legends).

### LC/MS analysis of CDP-Rbo and CDP-Rbo(TetA)

Mouse muscle samples were homogenized in ice-cold 75% ethanol, centrifuged at 10,000× *g* for 10 min at 4 °C, and the supernatant was dried under vacuum. Lipids were then removed via n-butanol extraction and metabolites were extracted using Envi-Carb graphitized carbon columns (250 mg; Merck Supelco) as described previously^42^. Briefly, samples were resuspended in 9% n-butanol, spiked with 5 pmol [^13^C]CDP-glycerol ([^13^C]CDP-Gro) (see below) as an internal standard to normalize CDP-Rbo recovery, and extracted twice with 90 % n-butanol. The resulting aqueous phase was dried under vacuum and dissolved in 5 mM ammonium bicarbonate. Envi-Carb columns were conditioned with 2.5 mL of 80% acetonitrile (MeCN) and 0.1% trifluoroacetic acid (TFA), followed by 2.5 mL of water. After each sample was applied to the column, it was sequentially washed with 2.5 mL of water, 2.5 mL of 25 % MeCN, and 2.5 mL of 50 mM triethylamine acetate (TEAA) buffer (pH 7) and eluted with 1.25 mL of 25% MeCN, 50 mM TEAA buffer (pH 7). The eluate was dried under vacuum and stored at −80 °C until further analysis.

Extracts were resuspended in 0.1 % formic acid (FA) adjusted to pH 9 with 1 M ammonia solution (mobile phase A) and metabolites were analyzed using porous graphitic carbon (PGC)-based LC-MS, as described previously^43^ with some modifications. Briefly, the metabolites were separated using a Hypercarb PGC column (3 μm, 2.1 × 50 mm; Thermo Fisher Scientific) with mobile phase A and mobile phase B, MeCN, under the following elution gradient: 0–1 min, 2% B; 1–16 min, 2–50% B; 16–17 min, 50–98% B; 17–18.5 min, 98% B; 18.5–19.5 min, 98–2% B; 19.5–30 min, 2% B. The column was maintained at 60 °C and a flow rate of 200 μL/min. MS analysis was performed using a Q-Exactive hybrid quadrupole-Orbitrap mass spectrometer (Thermo Fisher Scientific) operated in ESI + mode with selected ion monitoring (SIM) scanning and the following settings: spray voltage, 3.5 kV; sheath gas flow rate, 45 arbitrary units (a.u.); auxiliary gas flow rate, 10 a.u.; sweep gas flow rate, 2 a.u.; capillary temperature, 250 °C; S-lens RF level, 50; probe heater temperature, 400 °C; resolution, 70,000; automatic gain control target, 50,000; maximum IT, 200 ms; isolation window, 4.0 m/z. Each metabolite was quantified by calculating the peak area of the extracted ion chromatograms using a mass tolerance of 3 ppm ([M + H]^+^: CDP-Rbo, 538.0834; CDP-Rbo(TetA), 706.1256; [^13^C]CDP-Gro, 487.0924). CDP-Rbo and CDP-Rbo(TetA) were quantified by correlating the amounts of known metabolites using calibration curves. CDP-Rbo concentrations were normalized using [^13^C]CDP-Gro recovery and expressed as pmol/mg tissue.

For plasma stability analysis, mouse plasma (Rockland, Limerick, PA, USA) and sterile human plasma (Cosmo Bio, Tokyo, Japan) were incubated with 100 μM CDP-Rbo or its derivatives at 37 °C. Incubation was stopped by adding ethanol at a final concentration of 75 % and CDP-Rbo levels were measured using the same procedures.

### [^13^C]CDP-Gro preparation

[^13^C]CDP-Gro was synthesized as described previously^43^ with slight modifications. Briefly, His-tagged AQ1368, a glycerol-phosphate cytidylyltransferase from *Aquifex aeolicus*, was expressed in *E. coli* BL21(DE3), purified using nickel-chelate chromatography (HisTrap HP 5 mL, GE Healthcare), and dialyzed with 50 mM Tris-HCl (pH 8.6) containing 150 mM NaCl and 5 mM MgCl_2_. [^13^C]CDP-Gro was produced by heating 20 μL of solution containing 50 mM Tris-HCl (pH 8.6), 5 mM MgCl_2_, 6.25 μg His-AQ1368, 2.5 mM CTP (Cytidine-13C9; Merck Sigma), and 2.5 mM glycerol-3-phosphate (Merck Sigma) at 37 °C for 5 min. The product was separated using reverse-phase HPLC with a COSMOSIL 5C18-AR-II column (4.6 × 250 mm; Nacalai Tesque) and isocratic elution with 20 mM TEAA buffer (pH 7). Product elution was monitored by measuring the absorbance at 260 nm. [^13^C]CDP-Gro was then collected, dried under vacuum, and dissolved in water. [^13^C]CDP-Gro production was confirmed by LC-MS analysis, as described above.

### Statistical analysis

Data represent the mean ± SEM or are presented as scatter plots with the mean. All statistical analyses were performed using GraphPad Prism v.8.20 for Windows (GraphPad Software, San Diego, CA, USA). To compare in vivo data for two groups, individual means were compared using the Mann-Whitney *U* test and Welch’s *t* test. When comparing more than three groups, the Kruskal-Wallis test was performed with Dunn’s post-hoc test on selected pairs. Western blot band intensity was quantified using Multi Gauge v.3.2 software (Fujifilm, Tokyo, Japan) with Tukey’s multiple comparison test and Welch’s *t* test. CDP-Rbo level was analyzed using ANOVA with Tukey’s post hoc test. All statistical analyses were two-sided tests, and *p* values of ≤ 0.05 were considered statistically significant.

### Reporting summary

Further information on research design is available in the Nature Research Reporting Summary linked to this article.

## Supplementary information

Supplementary Information

Reporting Summary

## Data Availability

All data supporting the findings described in this manuscript are available in the article and in the Supplementary Information and from the corresponding author upon reasonable request. Source data are provided with this paper.

## Source data

Source Data
