# Supplementary material for: CDP-ribitol prodrug treatment ameliorates ISPD-deficient muscular dystrophy mouse model
Source: Nat Commun. 2022 Apr 14;13:1847. doi: 10.1038/s41467-022-29473-4 (PMC9010444; doi:10.1038/s41467-022-29473-4)
Supplement: Supplementary file 1 — Supplementary Information [file 41467_2022_29473_MOESM1_ESM.pdf]

CDP-ribitol prodrug treatment ameliorates *ISPD*-deficient muscular dystrophy mouse model

Hideki Tokuoka, Rieko Imae, Hitomi Nakashima, Hiroshi Manya, Chiaki Masuda, Shunsuke Hoshino, Kazuhiro Kobayashi, Dirk J. Lefeber, Riki Matsumoto, Takashi Okada, Tamao Endo, Motoi Kanagawa, and Tatsushi Toda

Supplementary Information

Supplementary Figures 1-9
Supplementary Tables 1-3

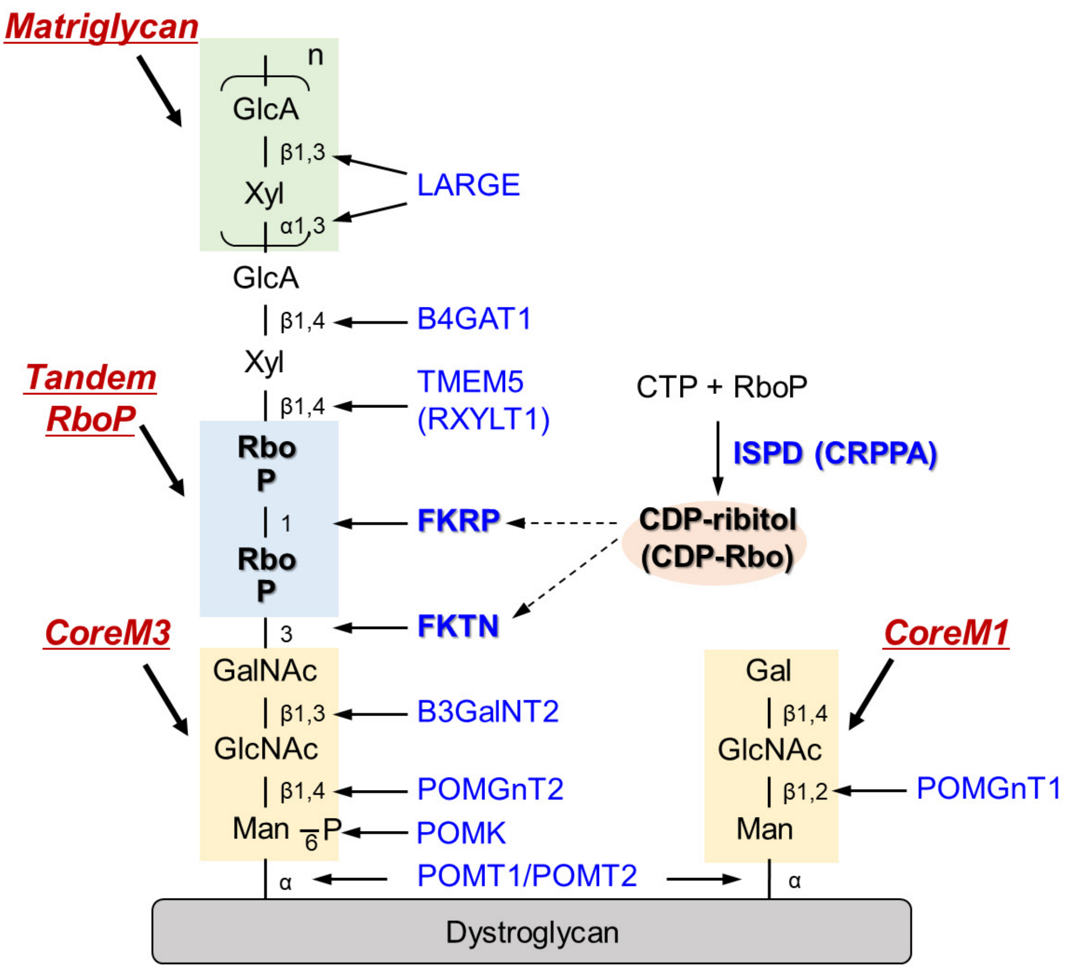

Supplementary Figure 1. Sugar chain structure of dystroglycan and enzymes responsible for glycosylation. Unique structural units (Matriglycan, Tandem RboP, CoreM3, and CoreM1) are indicated by colored boxes. Enzyme names are written in blue, and their functions are indicated by arrows. ISPD (CRPPA) synthesizes CDP-ribitol, a donor substrate for FKTN and FKRP. RboP, ribitol-phosphate. GlcA, glucuronic acid. Xyl, xylose. GalNAc, N-acetylgalactosamine. GlcNAc, N-acetylglucosamine. Man, mannose. Gal, galactose. P, phosphate.

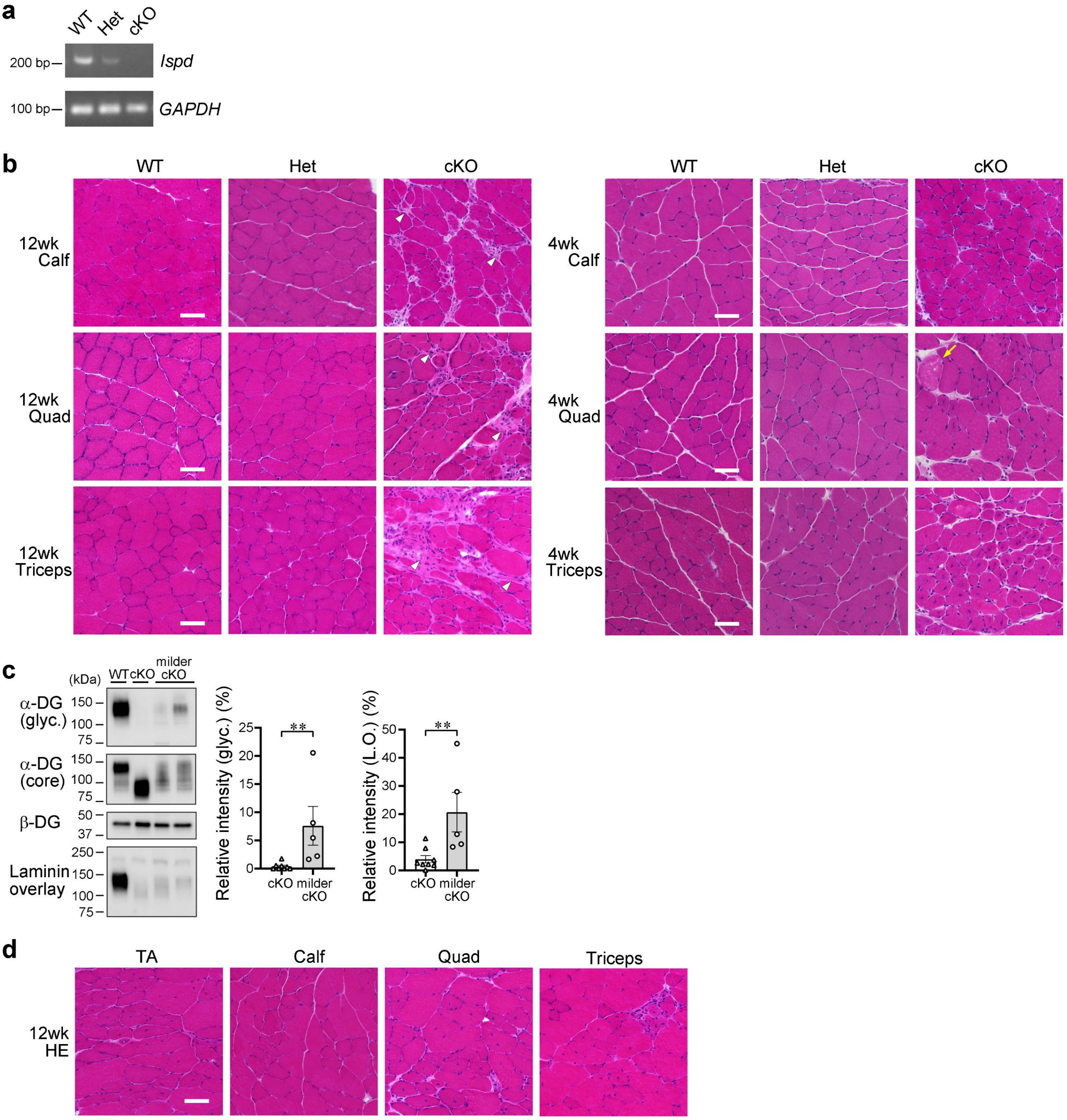

Supplementary Figure 2. RT-PCR and histopathological analysis of Myf5-*Ispd*-cKO mice, as well as biochemical analysis of Myf5-*Ispd*-cKO mice with a mild phenotype. (**a**) RT-PCR analysis of *Ispd* transcription. *GAPDH* was used as a control. The images are representative of three independent experiments. (**b**) Representative HE staining images of skeletal muscle from 4- and 12-week-old mice. Arrow, necrotic fiber; arrow head, fibrous connective tissue. The images are representative of at least four mice in each group. (**c**) Western blot analysis and laminin overlay assay of Myf5-*Ispd*-cKO mice with residual α-DG glycosylation. β-DG was used as a control. Right: relative matriglycan signal intensity (ratio of glyc./β-DG) and laminin-binding activity (ratio of laminin binding/β-DG) compared to those in WT muscle. All data represent the mean ± SEM. Data were analyzed using the Mann-Whitney *U* test (p = 0.003 and 0.006 for IIH6 and laminin overlay, respectively. cKO, n = 8; milder cKO, n = 5). (**d**) Representative HE staining image Myf5-*Ispd*-cKO mice with a mild phenotype. The images are representative of at least five mice in each group. Few myofibers with central nucleation and little fibrosis. Quad, quadriceps. L.O., laminin overlay. Scale bars, 50 μm.

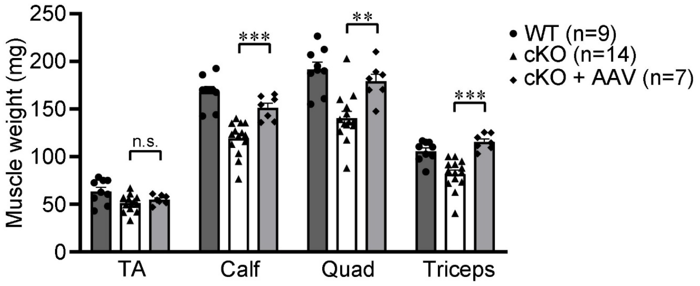

Supplementary Figure 3. Therapeutic effects of ISPD gene replacement in Myf5-*Ispd*-cKO mice (Muscle weight at 12 weeks of age). Four-week-old Myf5-Ispd cKO mice were injected with AAV9-MCK-ISPD (2 × 10^{12} v.g.) via the tail vein. After eight weeks, skeletal muscles were analyzed and compared to non-treated Myf5-*Ispd*-cKO mice (Calf, $p < 0.0001$; Quad, $p = 0.0015$; Triceps, $p < 0.0001$). Data were analyzed using the Mann-Whitney *U* test and represent the mean ± SEM. $^{**}p < 0.01$, and $^{***}p < 0.001$. Quad, quadriceps.

a

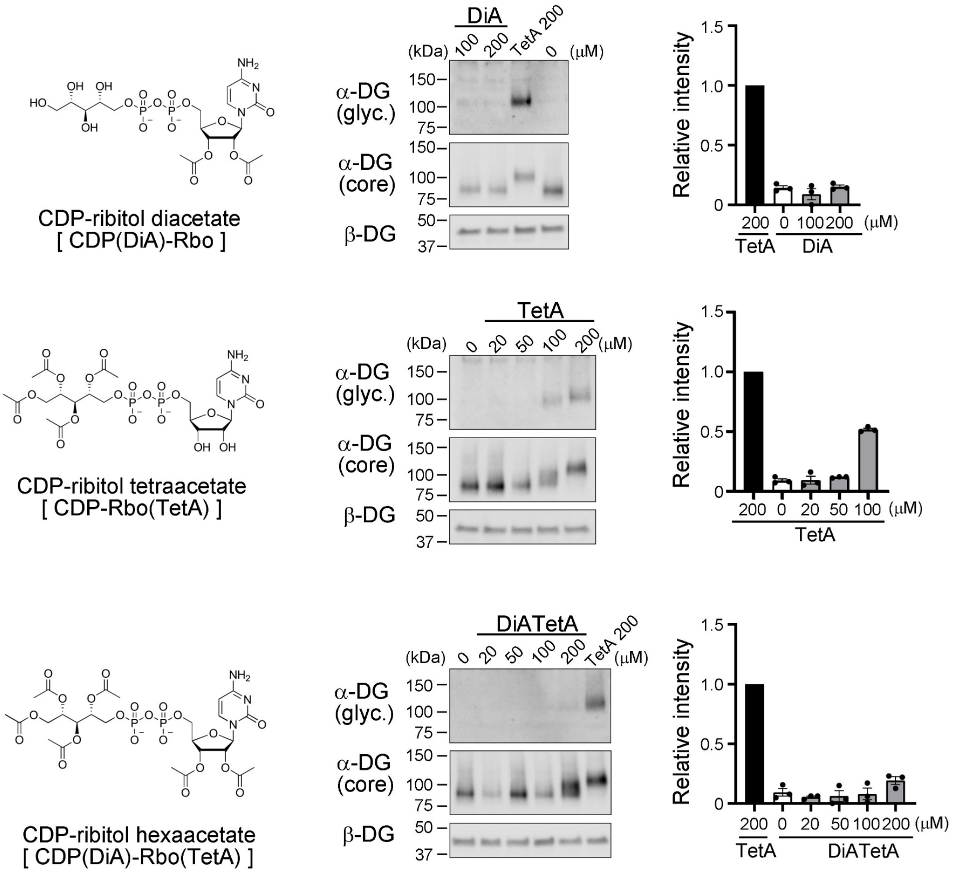

CDP-ribitol diacetate
[CDP(DiA)-Rbo]
DiA
100
200
TetA 200
0
(kDa)
(μM)
α-DG
(glyc.)
α-DG
(core)
β-DG
150
100
75
50
37
Relative intensity
1.5
1.0
0.5
0
200
TetA
0
100
200
(μM)
DiA
CDP-ribitol tetraacetate
[CDP-Rbo(TetA)]
TetA
0
20
50
100
200
CDP-ribitol hexaacetate
[CDP(DiA)-Rbo(TetA)]
DiATetA

b

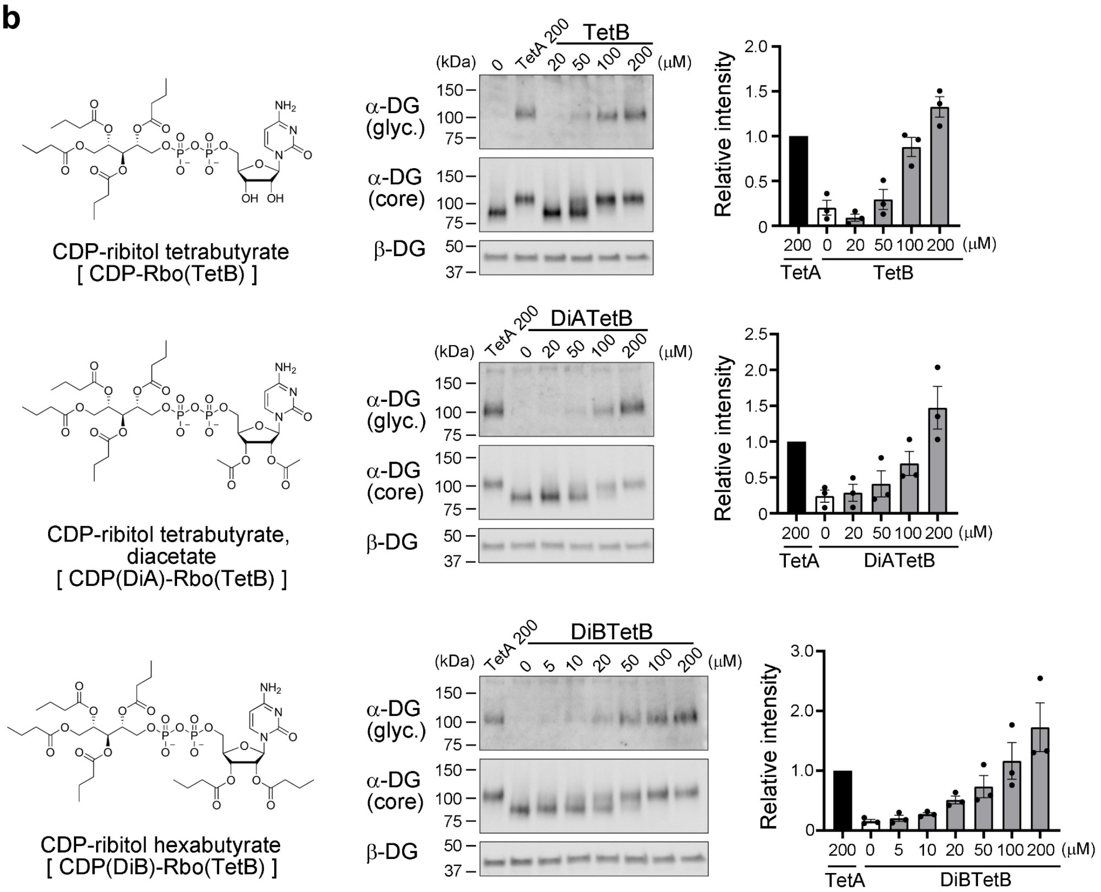

CDP-ribitol tetrabutyrate
[CDP-Rbo(TetB)]
TetB
TetA 200
(kDa)
(μM)
α-DG
(glyc.)
α-DG
(core)
β-DG
Relative intensity
2.0
1.5
1.0
0.5
0
CDP-ribitol tetrabutyrate,
diacetate
[CDP(DiA)-Rbo(TetB)]
DiATetB
2.5
CDP-ribitol hexabutyrate
[CDP(DiB)-Rbo(TetB)]
DiBTetB
3.0
0
5
10
20
50
100
200
TetA

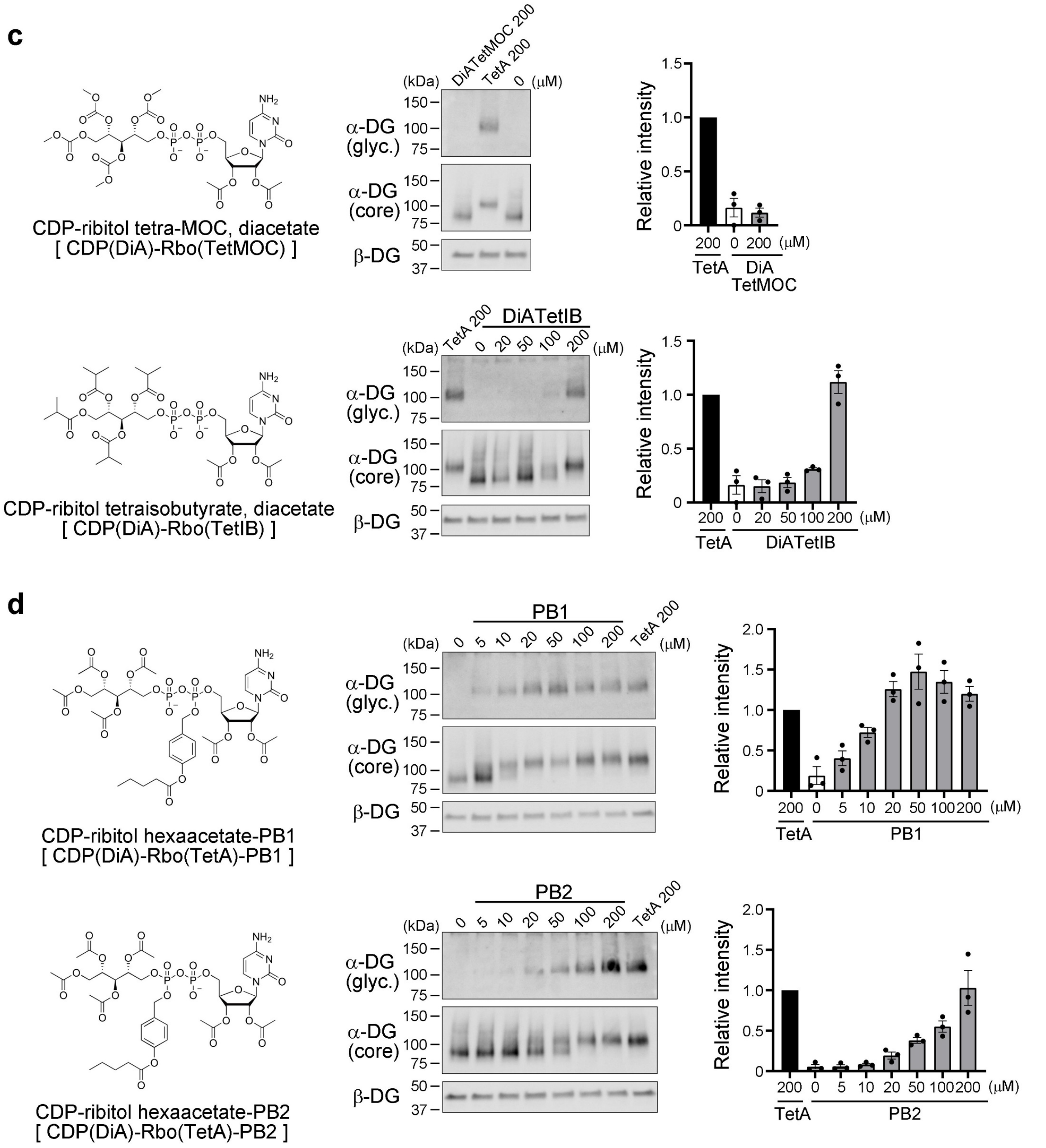

Supplementary Figure 4. CDP-Rbo derivative prodrug activities. Structural formula, nomenclature, and prodrug activity of CDP-Rbo acetylated (**a**), butylated (**b**), isobutylated or O-methoxycarbonylated (**c**), and pentanoyloxybenzylated (**d**) derivatives in *ISPD*-deficient HEK293 cells. Glycosylation status was measured using western blot analysis and shown as the relative matriglycan signal intensity (ratio of glyc./β-DG) compared to 200 μM CDP-Rbo(TetA)-treated cells. β-DG was used as the loading control. CDP-Rbo(TetA) (200 μM) was used as a positive control. All data represent the mean ± SEM (n = 3 per group). TetA, CDP-Rbo(TetA). DiA, CDP(DiA)-Rbo. DiATetA, CDP(DiA)-Rbo(TetA). TetB, CDP-Rbo(TetB). DiATetB, CDP(DiA)-Rbo(TetB). DiBTetB, CDP(DiB)-Rbo(TetB). DiATetMOC, CDP(DiA)-Rbo(TetMOC). DiATetIB, CDP(DiA)-Rbo(TetIB). PB1, CDP(DiA)-Rbo(TetA)-PB1. PB2, CDP(DiA)-Rbo(TetA)-PB2.

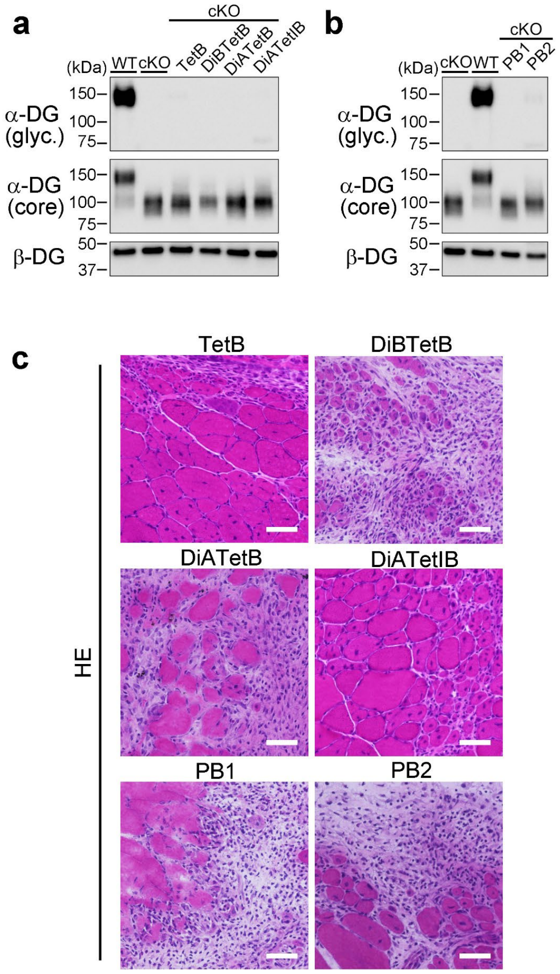

Supplementary Figure 5. Adverse effects of CDP-Rbo derivatives. Mice were injected with CDP-Rbo(TetB), CDP(DiB)-Rbo(TetB), CDP(DiA)-Rbo(TetB), CDP(DiA)-Rbo(TetIB), CDP(DiA)-Rbo(TetA)-PB1, and CDP(DiA)-Rbo(TetA)-PB2 twice weekly. (**a–b**) DG glycosylation in skeletal muscle. β-DG was used as a control. Lower CDP(DiB)-Rbo(TetB) (1/10), CDP(DiA)-Rbo(TetA)-PB1 (1/5) and CDP(DiA)-Rbo(TetA)-PB2 (1/5) concentrations were used compared to CDP-Rbo(TetA) due to high toxicity. (**c**) HE staining of tibialis anterior treated with CDP-Rbo derivatives. At least two mice were tested for each prodrug except DiBTetB and DiATetB (*n* = 1 each) given their severe toxicity. Scale bar, 50 μm. TetB, CDP-Rbo(TetB). DiATetB, CDP(DiA)-Rbo(TetB). DiBTetB, CDP(DiB)-Rbo(TetB). DiATetIB, CDP(DiA)-Rbo(TetIB). PB1, CDP(DiA)-Rbo(TetA)-PB1. PB2, CDP(DiA)-Rbo(TetA)-PB2.

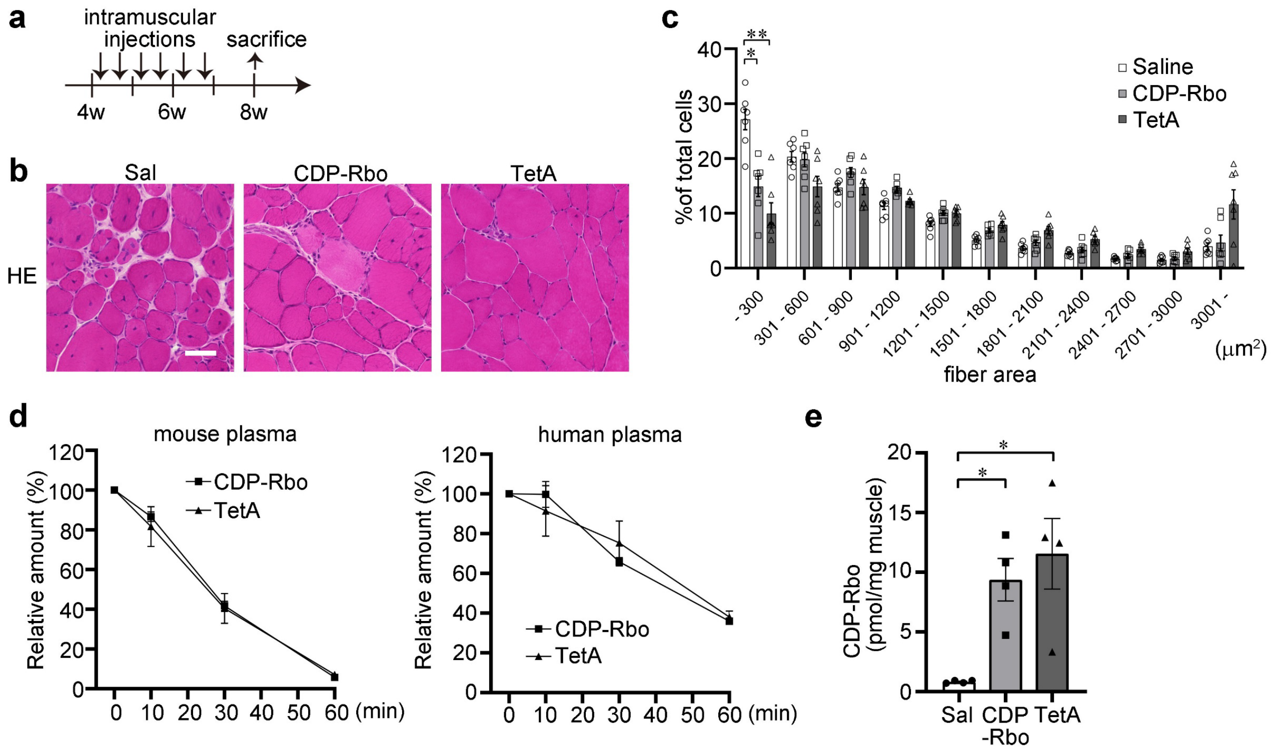

Supplementary Figure 6. Histopathological images, fiber size variation, and CDP-Rbo concentrations after CDP-Rbo(TetA) administration. (**a**) Schematic representation of long-term CDP-Rbo(TetA) treatment starting at 4 weeks of age. Saline-treated muscles were used as controls (Sal). (**b**) Representative HE staining images after CDP-Rbo and CDP-Rbo(TetA) administration. Scale bars, 50 μm. (**c**) Quantitative analysis of myofiber size variation (p = 0.037, Sal vs. CDP-Rbo; p = 0.001, Sal vs. CDP-Rbo(TetA) (fibers under 300 μm^2), n = 7). Data were analyzed using the Kruskal-Wallis ANOVA test followed by Dunn's multiple comparison. (**d**) CDP-Rbo and CDP-Rbo(TetA) stability in human or mouse plasma for the indicated time *in vitro* (n = 3). (**e**) CDP-Rbo concentration in WT skeletal muscle tissues 72 h after CDP-Rbo and CDP-Rbo(TetA) injection (p = 0.035, Sal vs. CDP-Rbo; p = 0.011, Sal vs. CDP-Rbo(TetA); n = 4). Data were analyzed using ANOVA with Tukey's post hoc test. All data represent the mean ± SEM. *p < 0.05 and **p < 0.01. TetA, CDP-Rbo(TetA).

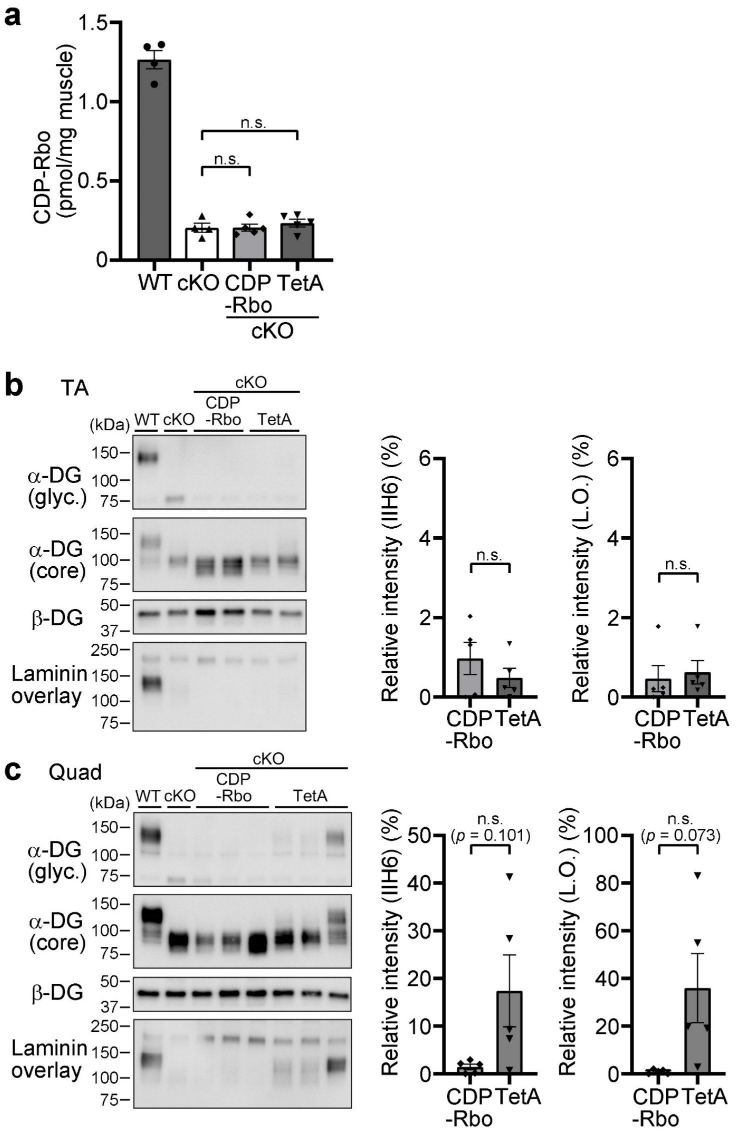

Supplementary Figure 7. Systemic CDP-Rbo prodrug administration does not restore α-DG glycosylation. WT or Myf5-*Ispd*-cKO mice were injected with CDP-Rbo or CDP-Rbo(TetA) twice a week via tail vein and subjected to biochemical analyses. (**a**) CDP-Rbo levels in skeletal muscle tissues (hamstrings) after systemic administration (WT, n = 4; cKO, n = 4; CDP-Rbo, n = 5; TetA, n = 5). **(b–c)** α-DG glycosylation measured using western blotting and laminin overlay analyses after CDP-Rbo or CDP-Rbo(TetA) administration. Right: relative matriglycan signal intensity (ratio of glyc./β-DG) and laminin-binding activity (ratio of laminin binding/β-DG) compared to those in WT muscle. All data represent the mean ± SEM. Data were analyzed using Welch's t test (**b**, tibialis anterior: **c**, Quadriceps, p = 0.101 and 0.073 for IIH6 and laminin overlay, respectively, n = 5). TA, tibialis anterior. Quad, quadriceps. TetA, CDP-Rbo(TetA). L.O., laminin overlay.

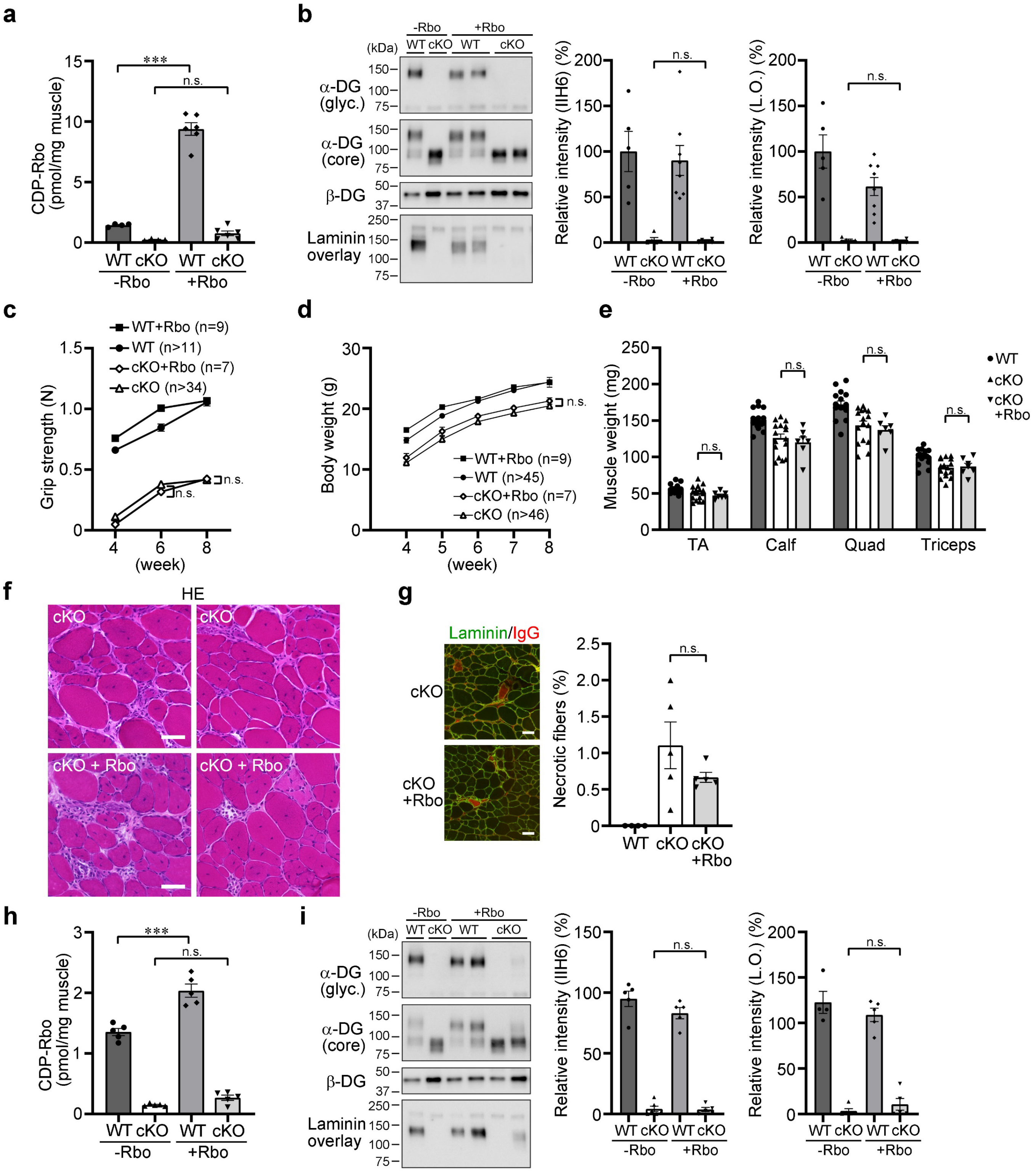
a
CDP-Rbo (pmol/mg muscle)
***
n.s.
WT cKO WT cKO
-Rbo +Rbo
b
-Rbo +Rbo
(kDa) WT cKO WT cKO
α-DG (glyc.)
α-DG (core)
β-DG
Laminin overlay
Relative intensity (IIH6) (%)
Relative intensity (L.O.) (%)
n.s.
c
WT+Rbo (n=9)
WT (n>11)
cKO+Rbo (n=7)
cKO (n>34)
Grip strength (N)
(week)
n.s.
d
Body weight (g)
WT+Rbo (n=9)
WT (n>45)
cKO+Rbo (n=7)
cKO (n>46)
(week)
n.s.
e
Muscle weight (mg)
WT
cKO
cKO +Rbo
TA Calf Quad Triceps
n.s.
f
HE
cKO
cKO
cKO + Rbo
cKO + Rbo
g
Laminin/IgG
cKO
cKO +Rbo
Necrotic fibers (%)
n.s.
WT cKO cKO +Rbo
h
CDP-Rbo (pmol/mg muscle)
***
n.s.
WT cKO WT cKO
-Rbo +Rbo
i
-Rbo +Rbo
(kDa) WT cKO WT cKO
α-DG (glyc.)
α-DG (core)
β-DG
Laminin overlay
Relative intensity (IIH6) (%)
Relative intensity (L.O.) (%)
n.s.

Supplementary Figure 8. Ribitol administration does not restore α-DG glycosylation or ameliorate the dystrophic pathology of Myf5-*Ispd*-cKO mice. **(a–g)** WT or Myf5-*Ispd*-cKO mice were administered with ribitol via drinking water for 4-weeks and subjected to biochemical and histopathological analyses. **(h–i)** TA and calf muscles were injected with ribitol twice a week and subjected to biochemical analyses. (**a**) CDP-Rbo levels in skeletal muscle tissues (hamstring) (p < 0.0001 for WT vs. WT + Rbo. -Rbo, n = 4 per group; +Rbo, n = 6 per group). **(b)** α-DG glycosylation measured using western blotting and laminin overlay analyses. Right: relative matriglycan signal intensity (ratio of glyc./β-DG) and laminin-binding activity (ratio of laminin binding/β-DG) compared to those in WT muscle (TA) (n = 5, WT - Rbo; n = 5, cKO - Rbo; n = 8, WT + Rbo; cKO + Rbo, n = 6). (**c–e**) Temporal changes in grip strength (**c**), body weight (**d**), muscle weight (**e**; WT, n = 16; cKO, n = 16; cKO + ribitol, n = 7). (**f**) Representative HE staining images after ribitol treatment (TA). (**g**) Immunofluorescence and quantitative analysis of necrotic fibers (TA) (WT; n = 4, cKO, n= 5, cKO + Rbo, n =5). (**h**) CDP-Rbo levels in skeletal muscle tissues (Calf) after intramuscular injections of ribitol (p < 0.0001 for WT vs. WT + Rbo, n = 5). (**i**) α-DG glycosylation after intramuscular injections of ribitol (TA). Right: relative matriglycan signal intensity (ratio of glyc./β-DG) and laminin-binding activity (ratio of laminin binding/β-DG) compared to those in WT muscle (IIH6: WT, n = 5; cKO, n = 6; WT + Rbo, n = 5; cKO + Rbo, n = 5. Laminin-binding: WT, n = 4; cKO, n = 5; WT + Rbo, n = 5; cKO + Rbo, n = 5). All data represent the mean ± SEM. CDP-Rbo level was analyzed using ANOVA with Tukey's post hoc test. Other data were analyzed using Welch's t test to compare cKO and cKO + Rbo. ***p < 0.001. Scale bars, 50 µm. Rbo, ribitol. TA, tibialis anterior. Quad, quadriceps. L.O., laminin overlay.

a. CDP-ribitol diacetate [CDP(DiA)-Rbo]

Chemical Nomenclature

(2R,3R,4R,5R)-2-(4-Amino-2-oxopyrimidin-1(2H)-yl)-5-(((hydroxy((hydroxy(((2R,3S,4S)-2,3,4,5-tetrahydroxypentyl)oxy)phosphoryl)oxy)phosphoryl)oxy)methyl)tetrahydrofuran-3,4-diyl diacetate

HPLC chart

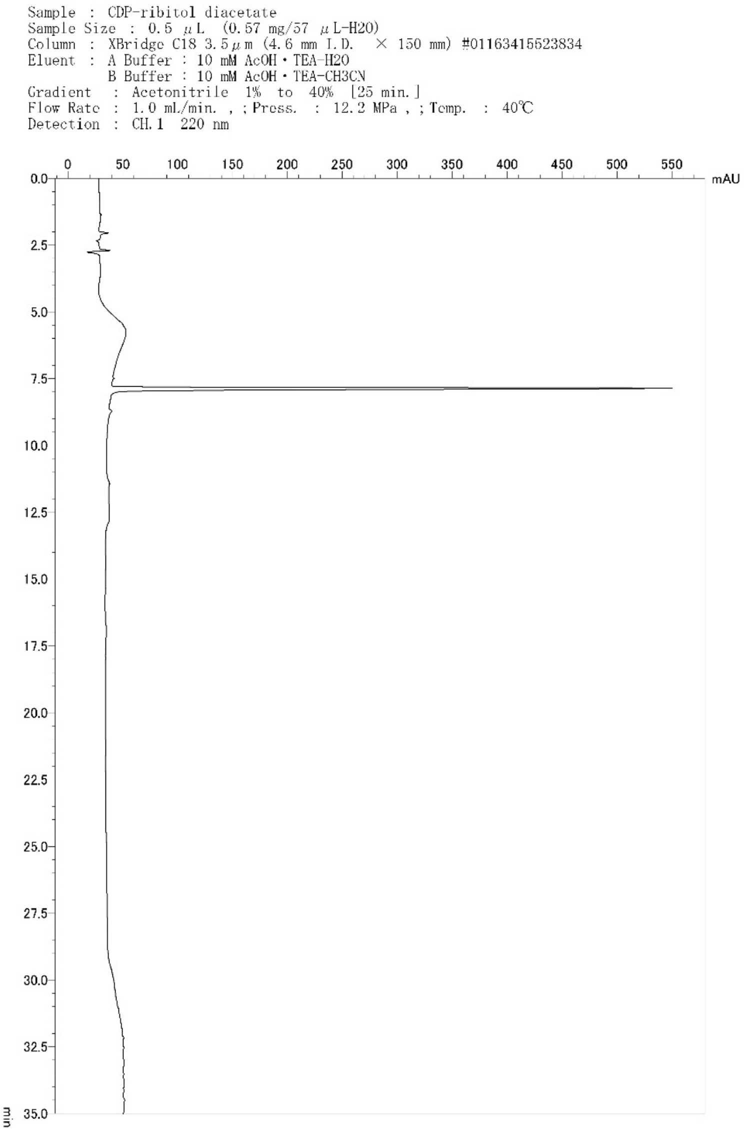

ESI-MS spectrum (negative mode)

$C_{18}H_{28}N_3O_{17}P_2$:[M-H]$^-$, calcd: 620.09, found: 620.1

^{1}H-NMR spectrum(400 MHz, in D_2O, ppm)

7.94(d, 1H, J=7.3 Hz), 6.22(d, 1H, J=5.5 Hz), 6.15(d, 1H, J=7.8 Hz), 5.48(dd, 1H, J=4.1 and 5.5 Hz), 5.44(dd, 1H, J=5.5 and 5.5 Hz), 4.53(m, 1H), 4.29(ddd, 1H, J=2.3, 5.0 and 11.9 Hz), 4.21(ddd, 1H, J=3.2, 5.0 and 11.9 Hz), 4.15(ddd, 1H, J=3.0, 5.7 and 11.0 Hz), 4.06(m, 1H), 3.91(m, 1H), 3.87(m, 1H), 3.80(dd, 1H, J=3.2 and 11.9 Hz), 3.76(dd, 1H, J=5.9 and 6.9 Hz), 3.64(dd, 1H, J=6.9 and 11.9 Hz), 2.18(s, 3H), 2.12(s, 3H)

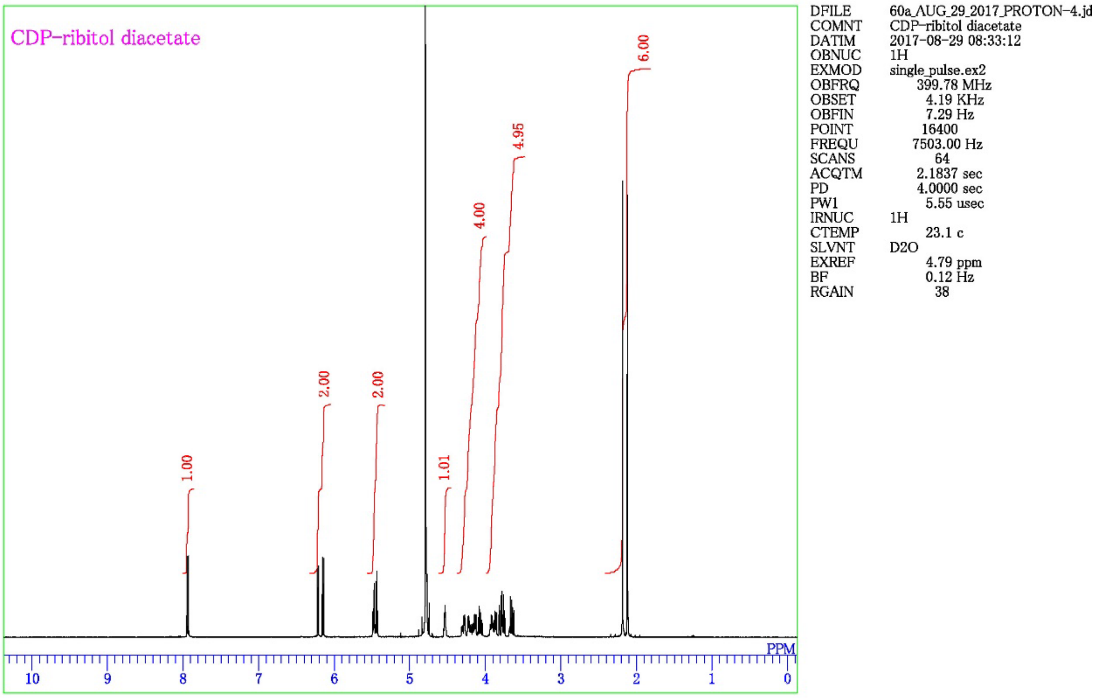

b. CDP-ribitol tetraacetate [CDP-Rbo(TetA)]

Chemical Nomenclature

(2S,3S,4R)-5-(((((((2R,3S,4R,5R)-5-(4-Amino-2-oxopyrimidin-1(2H)-yl)-3,4-dihydroxytetrahydrofuran-2-yl)methoxy)(hydroxy)phosphoryl)oxy)(hydroxy)phosphoryl)oxy)pentane-1,2,3,4-tetrayl tetraacetate

HPLC chart

Sample : CDP-ribitol tetraacetate
Sample Size : 1.0 μL(1.27 mg/127 μL-H2O)
Column : XBridge C18 3.5um (4.6 mm I.D. × 150 mm) #01803612413836
Eluent : A Buffer:10 mM AcOH・TEA-H2O
B Buffer:10 mM AcOH・TEA-CH3CN
Gradient : Acetonitrile 10% to 60% [25 min.]
Flow Rate : 1.0 mL/min. , ; Press. : 16 MPa , ; Temp. : 40℃
Detection : CH.1 220 nm

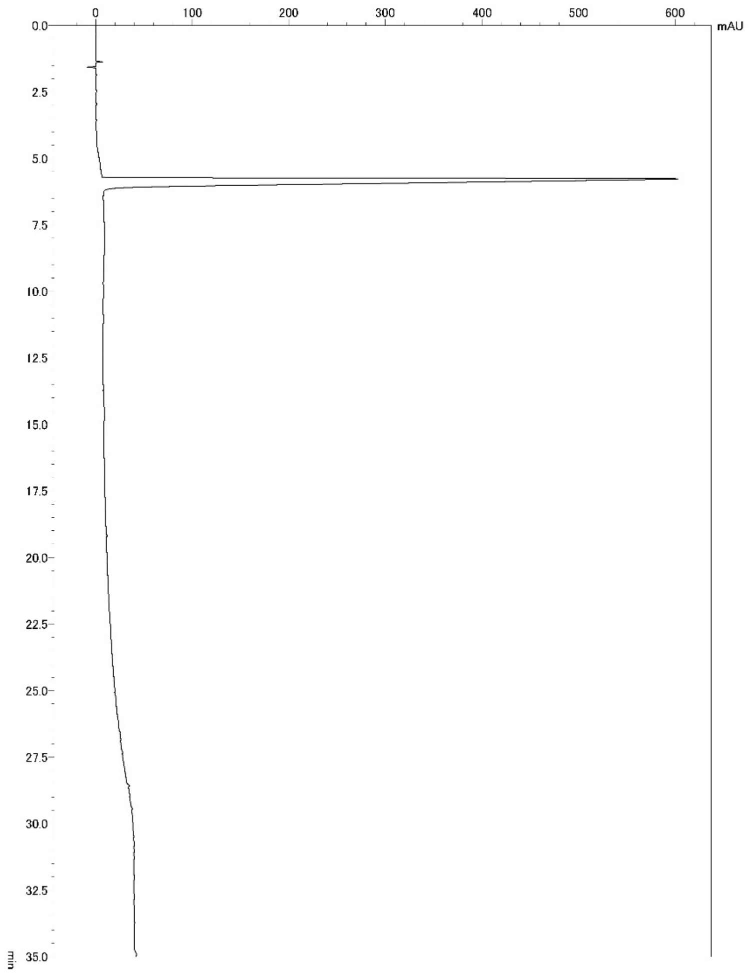

ESI-MS spectrum (negative mode)

$C_{22}H_{32}N_3O_{19}P_2$:[M-H]$^-$, calcd: 704.11, found: 704.0

^{1}H-NMR spectrum(400 MHz, in D_2O, ppm)

8.00(d, 1H, J=7.3 Hz), 6.12(d, 1H, J=7.3 Hz), 5.97(d, 1H, J=4.1 Hz), 5.36(dd, 1H, J=5.5 and 5.5 Hz), 5.31-5.26(m, 2H), 4.40(dd, 1H, J=3.2 and 12.4 Hz), 4.35-4.23(m, 5H), 4.20-4.15(m, 2H), 4.06(m, 1H), 2.129(s, 3H), 2.118(s, 3H), 2.118(s, 3H), 2.071(s, 3H)

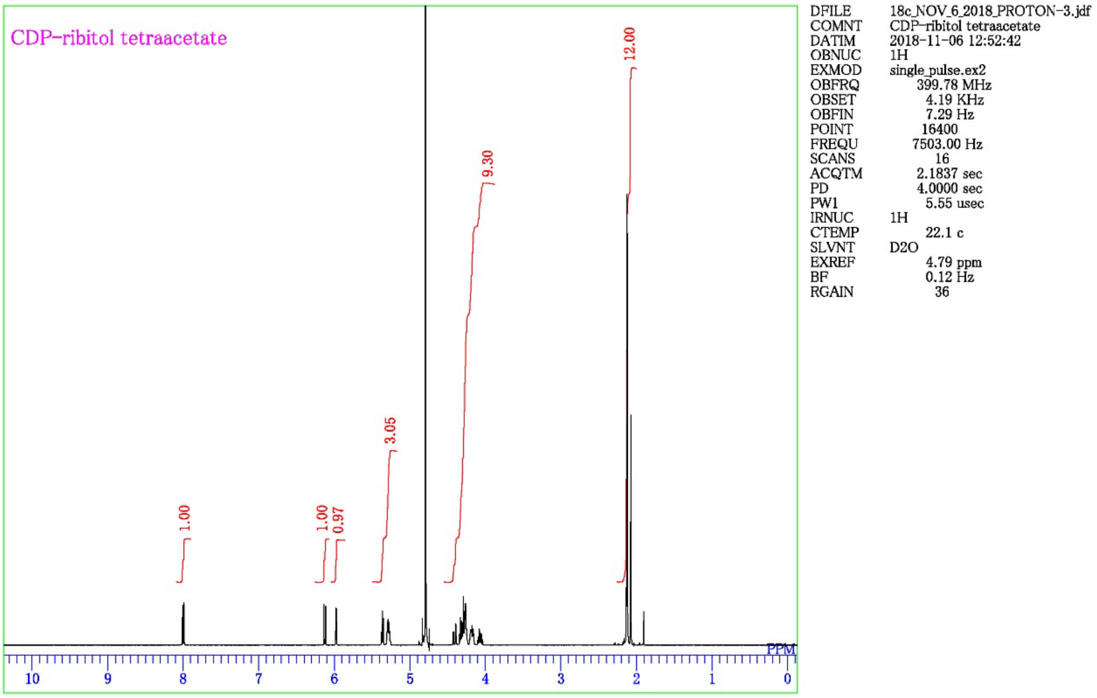

c. CDP-ribitol hexaacetate [CDP(DiA)-Rbo(TetA)]

Chemical Nomenclature

(2S,3S,4R)-5-(((((((2R,3S,4R,5R)-5-(4-Amino-2-oxopyrimidin-1(2H)-yl)-3,4-dihydroxytetrahydrofuran-2-yl)methoxy)(hydroxy)phosphoryl)oxy)(hydroxy)phosphoryl)oxy)pentane-1,2,3,4-tetrayl tetraacetate

HPLC chart

Sample : CDP-ribitol hexaacetate
Sample Size : 1.0 μL(0.63 mg/63 μL-H2O)
Column : XBridge C18 3.5um (4.6 mm I.D. × 150 mm) #01803612413836
Eluent : A Buffer:10 mM AcOH・TEA-H2O
B Buffer:10 mM AcOH・TEA-CH3CN
Gradient : Acetonitrile 10% to 60% [25 min.]
Flow Rate : 1.0 mL/min. , ; Press. : 15.5 MPa , ; Temp. : 40℃
Detection : CH.1 220 nm

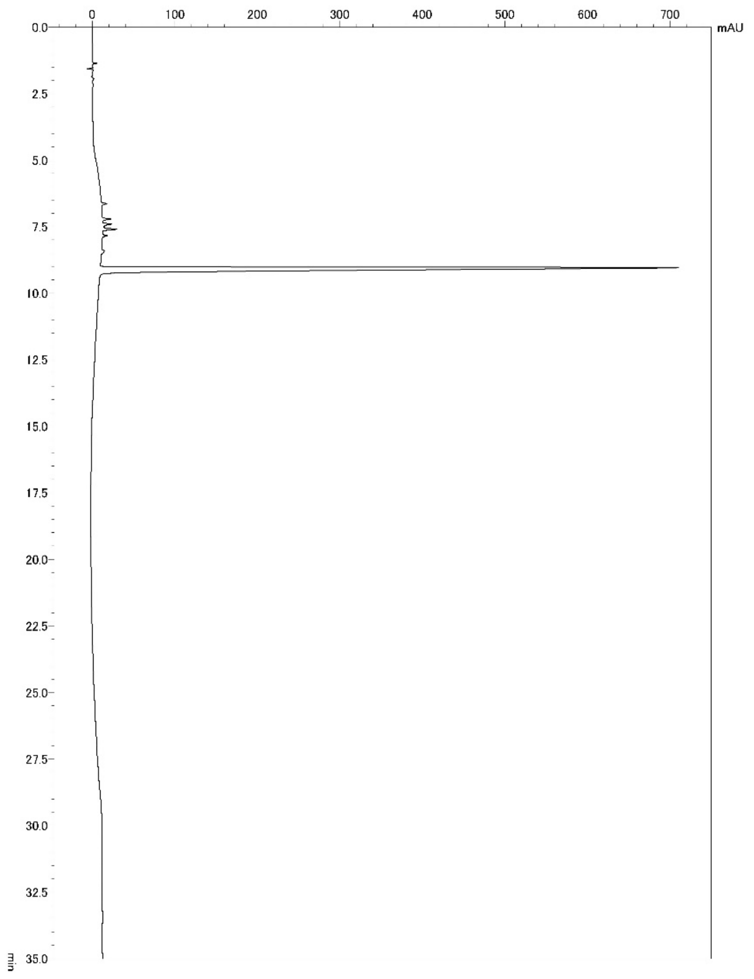

ESI-MS spectrum (negative mode)

$C_{26}H_{36}N_3O_{21}P_2$:[M-H]$^-$, calcd: 788.13, found: 788.1

^{1}H-NMR spectrum(400 MHz, in D_2O, ppm)

7.97(d, 1H, J=7.3 Hz), 6.22(d, 1H, J=5.5 Hz), 6.16(d, 1H, J=7.3 Hz), 5.46(dd, 1H, J=4.1 and 5.5 Hz), 5.41(dd, 1H, J=5.5 and 5.5 Hz), 5.36(dd, 1H, J=5.5 and 5.5 Hz), 5.31-5.26(m, 2H), 4.52(m, 1H), 4.41(dd, 1H, J=3.2 and 12.4 Hz), 4.31-4.26(m, 2H), 4.20-4.16(m, 2H), 4.07(m, 1H), 2.18(s, 3H), 2.14(s, 3H), 2.13(s, 3H), 2.120(s, 3H), 2.118(s, 3H), 2.08(s, 3H)

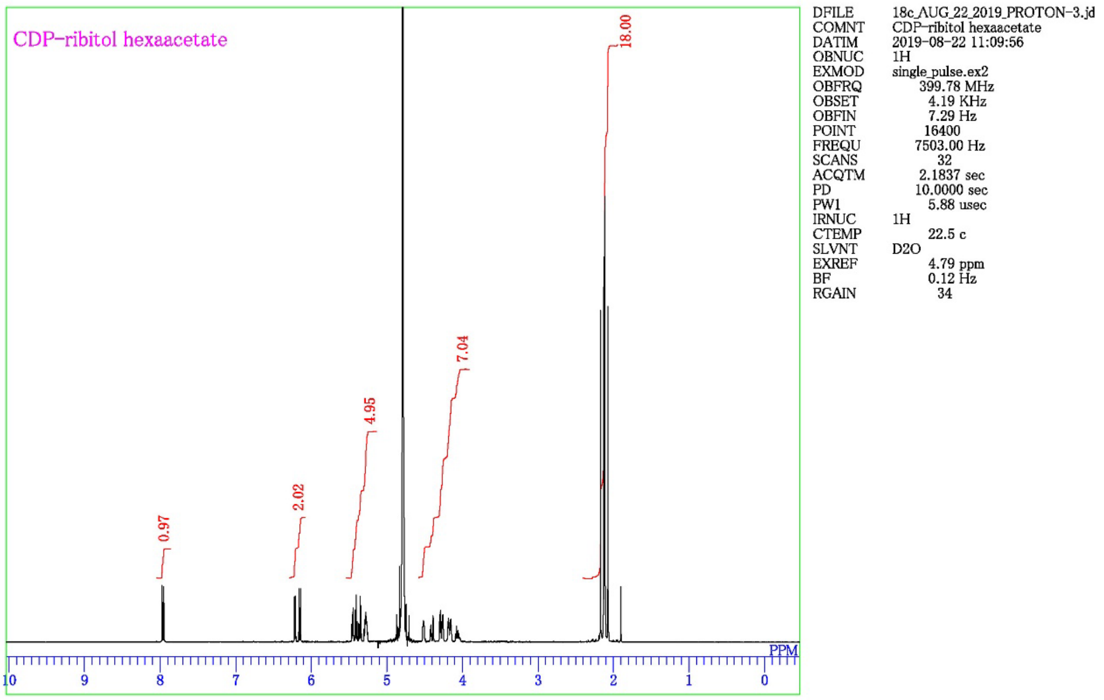

d. CDP-ribitol tetrabutyrate [CDP-Rbo(TetB)]

Chemical Nomenclature

(2S,3S,4R)-5-(((((((2R,3S,4R,5R)-5-(4-Amino-2-oxopyrimidin-1(2H)-yl)-3,4-dihydroxytetrahydrofuran-2-yl)methoxy)(hydroxy)phosphoryl)oxy)(hydroxy)phosphoryl)oxy)pentane-1,2,3,4-tetrayl tetrabutyrate

HPLC chart

Sample : CDP-ribitol tetrabutyrate (Na塩)
Sample Size : 0.8 μL(0.93 mg/93 μL-H2O)
Column : XBridge C18 3.5um (4.6 mm I.D. × 150 mm) #01663415523834
Eluent : A Buffer:10 mM AcOH・TEA-H2O
B Buffer:10 mM AcOH・TEA-CH3CN
Gradient : Acetonitrile 20% to 70% [25 min.]
Flow Rate : 1.0 mL/min. , ; Press. : 17.5 MPa , ; Temp. : 40℃
Detection : CH.1 220 nm

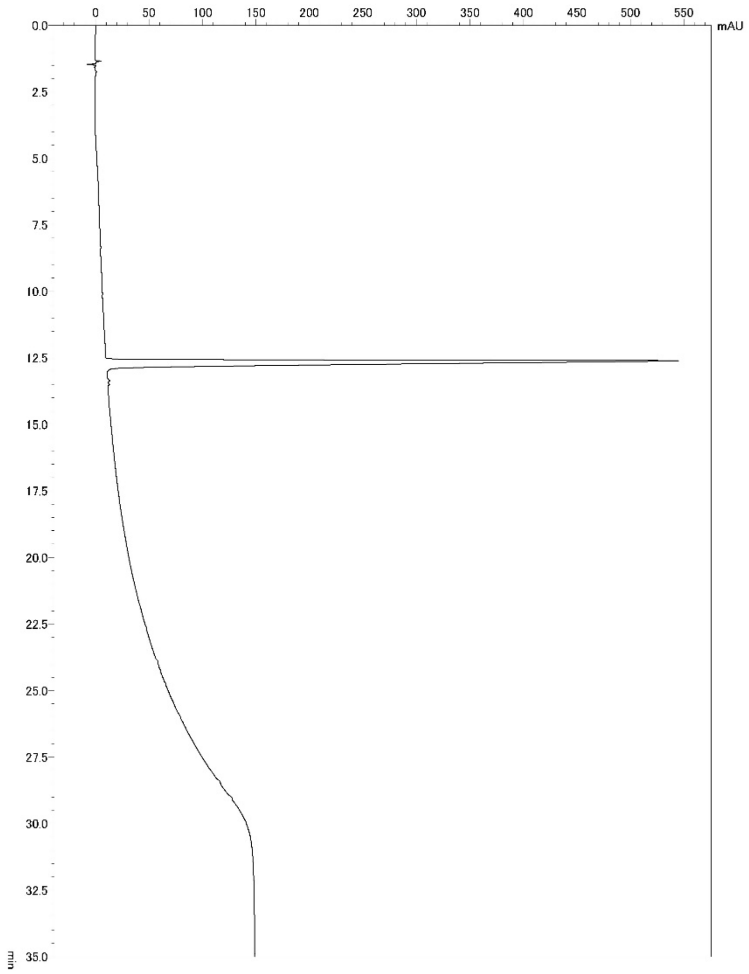

ESI-MS spectrum (negative mode)

$C_{30}H_{48}N_3O_{19}P_2$:[M-H]$^-$, calcd: 816.24, found: 816.2

^{1}H-NMR spectrum(400 MHz, in D_2O, ppm)

8.02(d, 1H, J=7.3 Hz), 6.13(d, 1H, J=7.3 Hz), 5.96(d, 1H, J=3.7 Hz), 5.39(dd, 1H, J=4.6 and 5.9 Hz), 5.31(m, 2H), 4.48(dd, 1H, J=2.7 and 12.4 Hz), 4.33-4.15(m, 7H), 4.05(ddd, 1H, J=6.4, 6.4, 11.4 Hz), 2.40-2.32(m, 8H), 1.65-1.54(m, 8H), 0.91(br.t, 3H+3H+3H, J=7.3 Hz), 0.89(t, 3H, J=7.3 Hz)

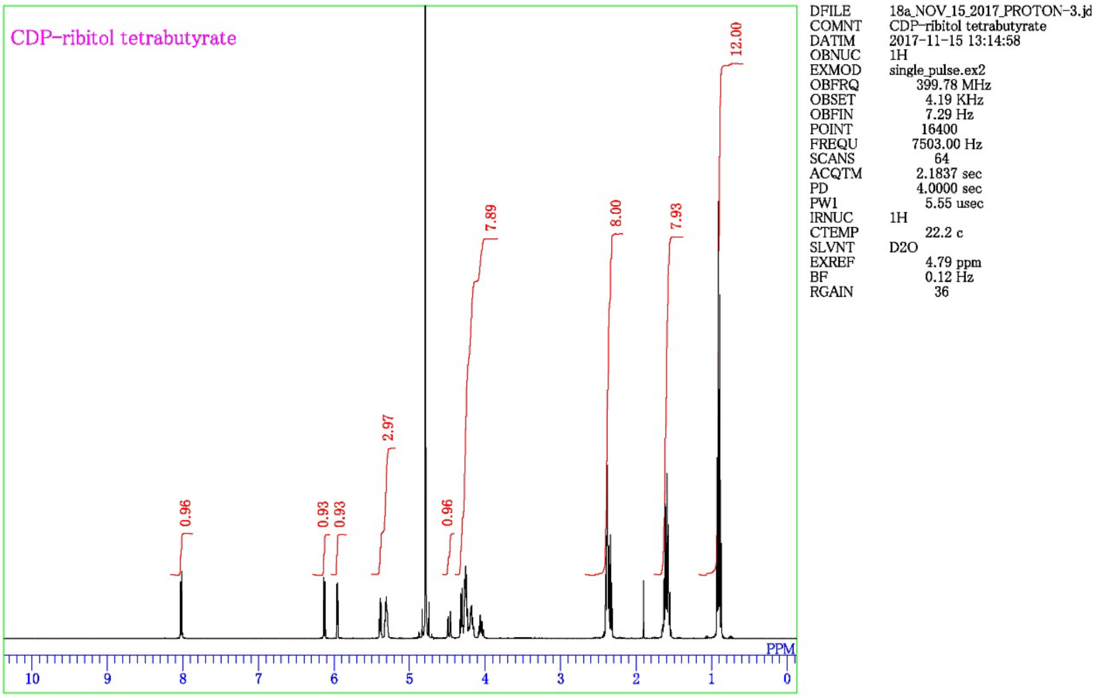

e. CDP-ribitol tetrabutyrate, diacetate [CDP(DiA)-Rbo(TetB)]

Chemical Nomenclature

(2S,3S,4R)-5-(((((((2R,3R,4R,5R)-3,4-Diacetoxy-5-(4-amino-2-oxopyrimidin-1(2H)-yl)tetrahydrofuran-2-yl)methoxy)(hydroxy)phosphoryl)oxy)(hydroxy)phosphoryl)oxy)pentane-1,2,3,4-tetrayl tetrabutyrate

HPLC chart

Sample : CDP-ribitol tetrabutyrate, diacetate (Na塩)
Sample Size : 1.0 μL(0.70 mg/70 μL-H2O)
Column : XBridge C18 3.5um (4.6 mm I.D. × 150 mm) #01663415523834
Eluent : A Buffer:10 mM AcOH・TEA-H2O
B Buffer:10 mM AcOH・TEA-CH3CN
Gradient : Acetonitrile 20% to 70% [25 min.]
Flow Rate : 1.0 mL/min. , ; Press. : 17.5 MPa , ; Temp. : 40℃
Detection : CH.1 220 nm

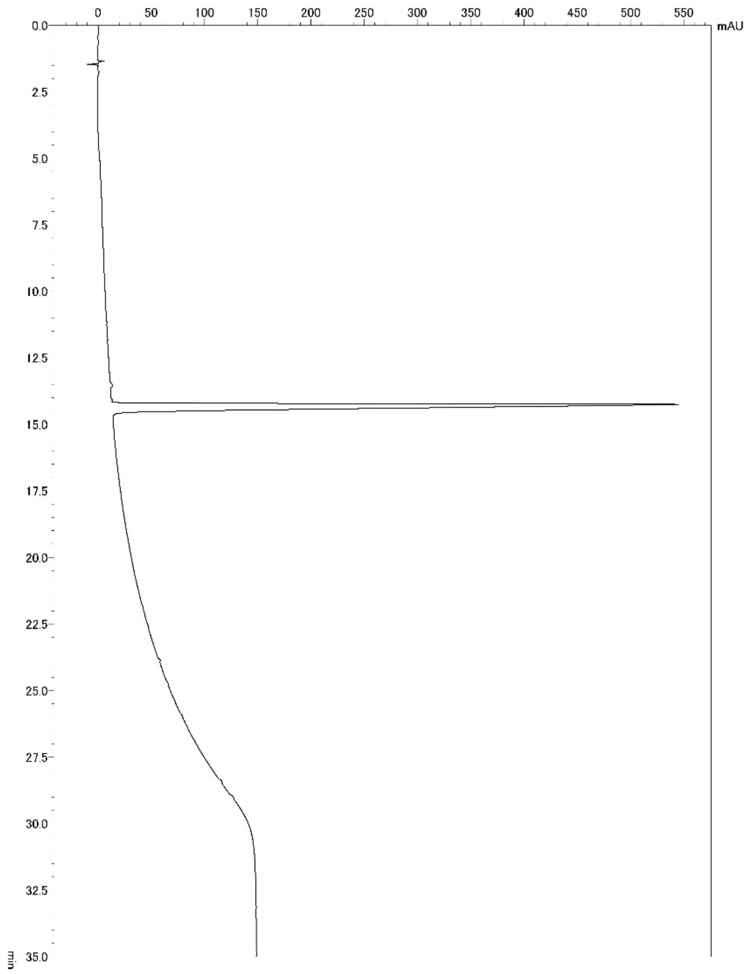

ESI-MS spectrum (negative mode)

$C_{34}H_{52}N_3O_{21}P_2$:[M-H]$^-$, calcd: 900.26, found: 900.3

^{1}H-NMR spectrum(400 MHz, in D_2O, ppm)

7.99(d, 1H, J=7.3 Hz), 6.21(d, 1H, J=5.5 Hz), 6.16(d, 1H, J=7.3 Hz), 5.45(dd, 1H, J=4.1 and 5.0 Hz), 5.41-5.37(m, 2H), 5.32-5.29(m, 2H), 4.51(m, 1H), 4.47(dd, 1H, J=2.3 and 12.4 Hz), 4.31-4.15(m, 4H), 4.05(ddd, 1H, J=5.9, 5.9 and 11.9 Hz), 2.41-2.32(m, 8H), 2.17(s, 3H), 2.12(s, 3H), 1.65-1.54(m, 8H), 0.91 (br.t, 3H+3H+3H, J=7.3 Hz), 0.89 (t, 3H, J=7.3 Hz)

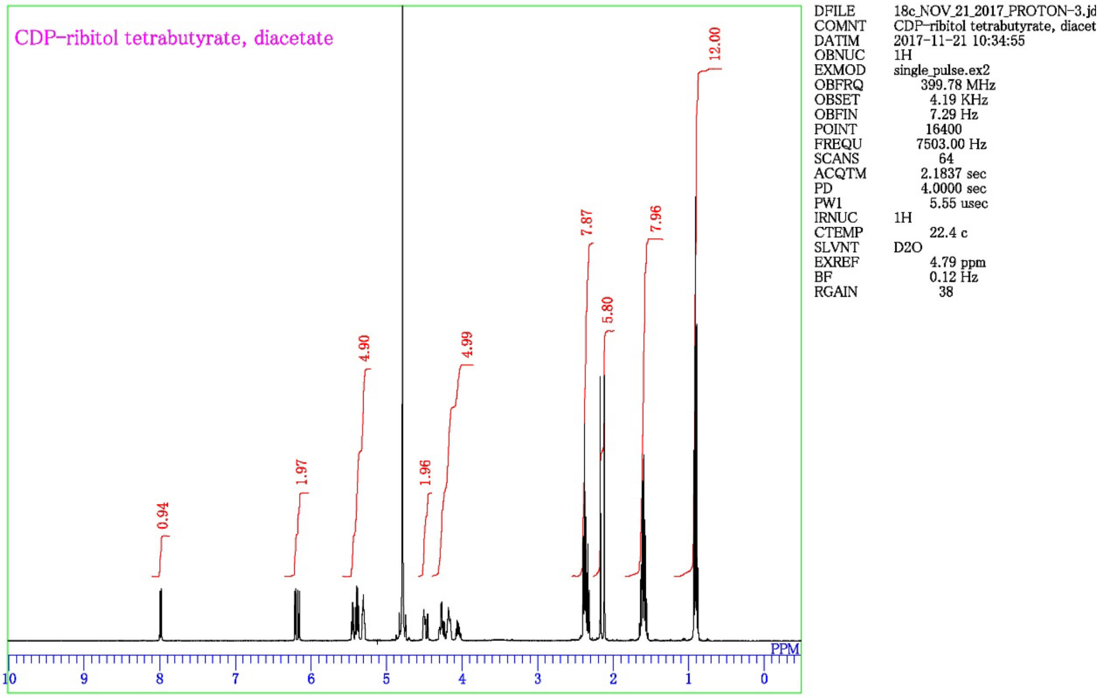

f. CDP-ribitol hexabutyrate [CDP(DiB)-Rbo(TetB)]

Chemical Nomenclature

(2S,3S,4R)-5-(((((((2R,3R,4R,5R)-5-(4-Amino-2-oxopyrimidin-1(2H)-yl)-3,4-bis(butyryloxy)tetrahydrofuran-2-yl)methoxy)(hydroxy)phosphoryl)oxy)(hydroxy)phosphoryl)oxy)pentane-1,2,3,4-tetrayl tetrabutyrate

HPLC chart

Sample : CDP-ribitol hexabutyrate (Na塩)
Sample Size : 1.0 μL(0.71 mg/71 μL-H2O)
Column : XBridge C18 3.5um (4.6 mm I.D. × 150 mm) #01663415523834
Eluent : A Buffer:10 mM AcOH・TEA-H2O
B Buffer:10 mM AcOH・TEA-CH3CN
Gradient : Acetonitrile 20% to 70% [25 min.]
Flow Rate : 1.0 mL/min. , ; Press. : 17.5 MPa , ; Temp. : 40℃
Detection : CH.1 220 nm

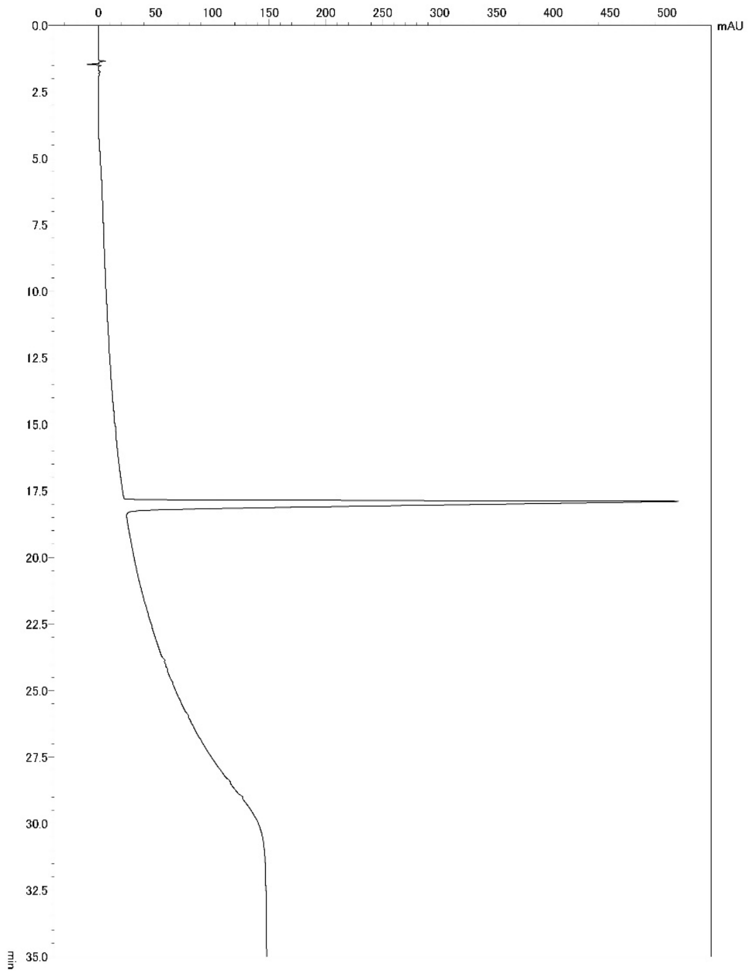

ESI-MS spectrum (negative mode)

$C_{38}H_{60}N_3O_{21}P_2$:[M-H]$^-$, calcd: 956.32, found: 956.3

^{1}H-NMR spectrum(400 MHz, in D_2O, ppm)

7.99(d, 1H, J=7.8 Hz), 6.21(d, 1H, J=5.9 Hz), 6.16(d, 1H, J=7.8 Hz), 5.48(m, 1H), 5.42(m, 1H), 5.38(m, 1H), 5.34-5.28(m, 2H), 4.50-4.45(m, 2H), 4.30-4.24(m, 2H), 4.21-4.15(m, 2H), 4.05(ddd, 1H, J=5.9, 5.9 and 11.9 Hz), 2.45(t, 2H, J=7.3 Hz), 2.40-2.32(m, 10H), 1.70-1.54(m, 12H), 0.97-0.86(m, 18H)

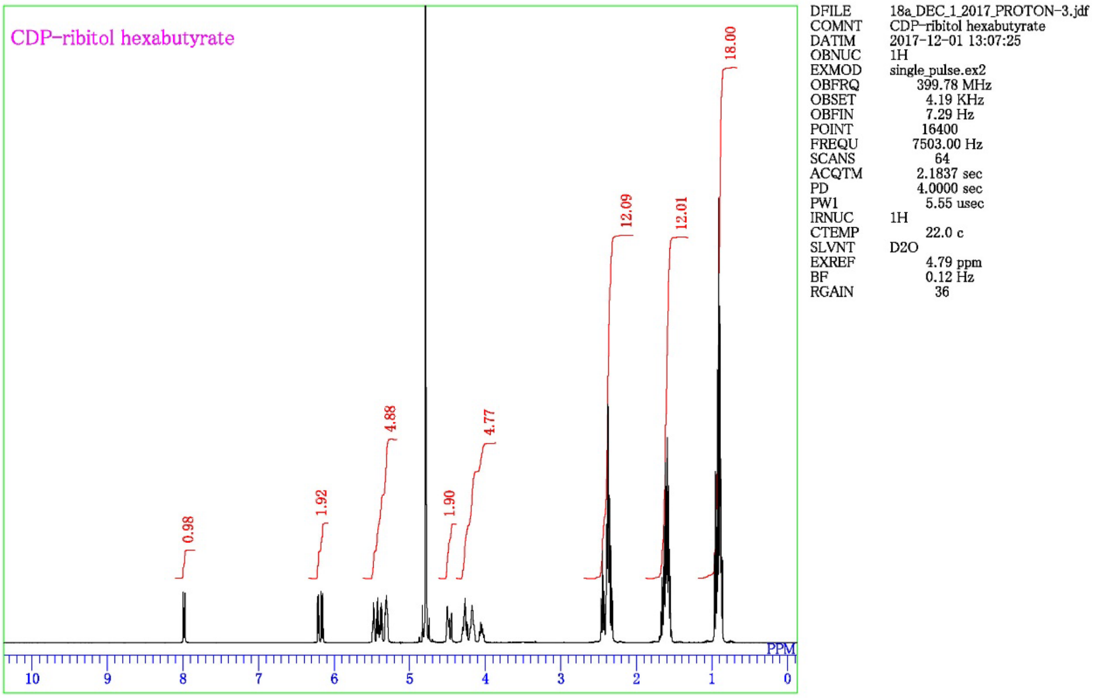

g. CDP-ribitol tetraisobutyrate, diacetate [CDP(DiA)-Rbo(TetIB)]

Chemical Nomenclature

(2S,3S,4R)-5-(((((((2R,3R,4R,5R)-3,4-Diacetoxy-5-(4-amino-2-oxopyrimidin-1(2H)-yl)tetrahydrofuran-2-yl)methoxy)(hydroxy)phosphoryl)oxy)(hydroxy)phosphoryl)oxy)pentane-1,2,3,4-tetrayl tetrakis(2-methylpropanoate)

HPLC chart

Sample : CDP-ribitol tetraisobutyrate, diacetate
Sample Size : 1.0 μL(1.07 mg/107 μL-H2O)
Column : XBridge C18 3.5um (4.6 mm I.D. × 150 mm) #01663415523834
Eluent : A Buffer:10 mM AcOH・TEA-H2O
B Buffer:10 mM AcOH・TEA-CH3CN
Gradient : Acetonitrile 20% to 70% [25 min.]
Flow Rate : 1.0 mL/min. , ; Press. : 17 MPa , ; Temp. : 40℃
Detection : CH.1 220 nm

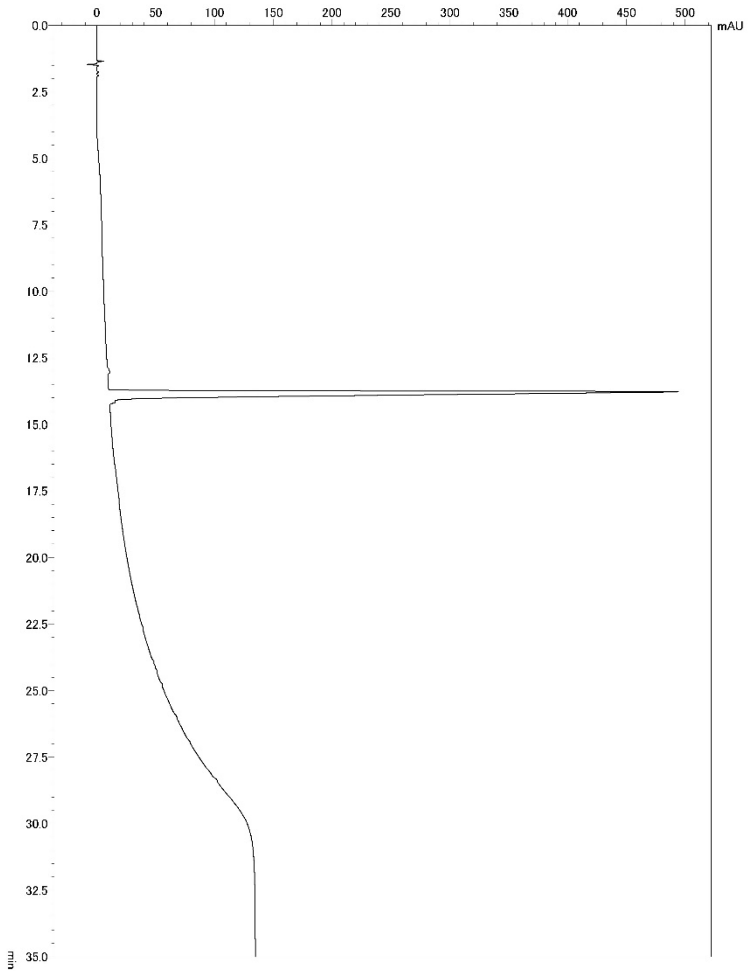

ESI-MS spectrum (negative mode)

$C_{34}H_{52}N_3O_{21}P_2$:[M-H]$^-$, calcd: 900.26, found: 900.2

^{1}H-NMR spectrum(400 MHz, in D_2O, ppm)

8.00(d, 1H, J=7.8 Hz), 6.20(d, 1H, J=5.5 Hz), 6.16(d, 1H, J=7.3 Hz), 5.44(dd, 1H, J=4.6 and 5.5 Hz), 5.41-5.37(m, 2H), 5.31-5.26(m, 2H), 4.52-4.46(m, 2H), 4.30-4.13(m, 4H), 4.06(ddd, 1H, J=6.9, 6.9 and 11.4 Hz), 2.68-2.54(m, 4H), 2.16(s, 3H), 2.11(s, 3H), 1.15-1.12(m, 18H), 1.11(d, 3H, J=6.9 Hz), 1.10(d, 3H, J=6.9 Hz)

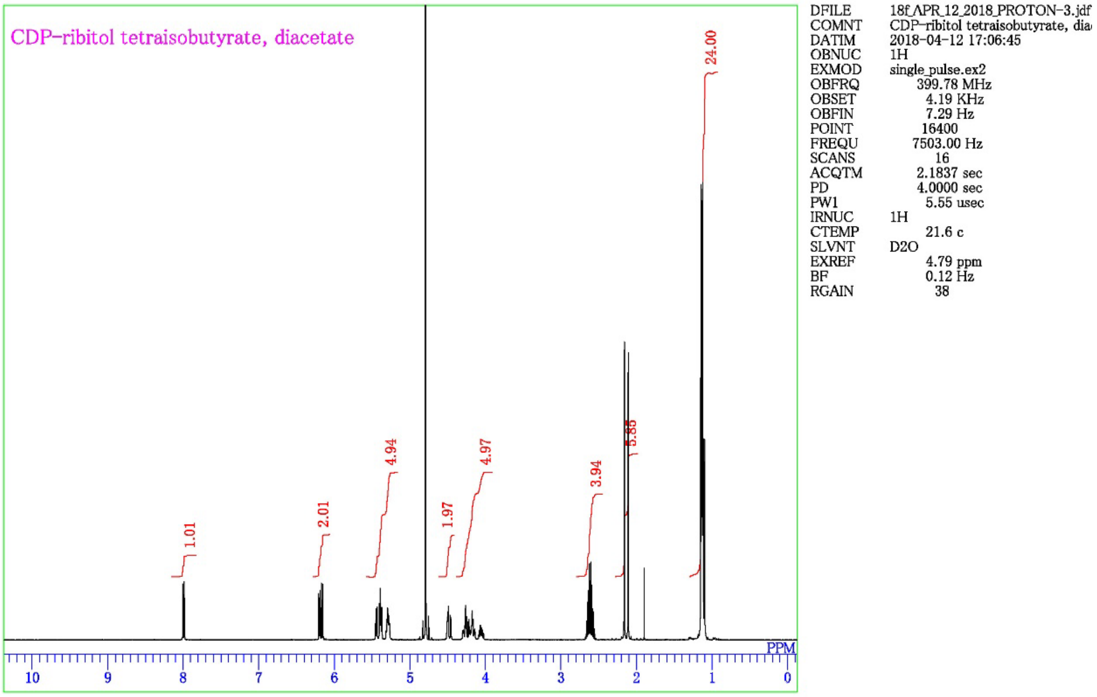

h. CDP-ribitol tetra-Moc, diacetate [CDP(DiA)-Rbo(TetMOC)]

Chemical Nomenclature

(2R,3R,4R,5R)-2-(4-Amino-2-oxopyrimidin-1(2H)-yl)-5-(((hydroxy((hydroxy(((2R,3S,4S)-2,3,4,5-tetrakis((methoxycarbonyl)oxy)pentyl)oxy)phosphoryl)oxy)phosphoryl)oxy)methyl)tetrahydrofuran-3,4-diyl diacetate

HPLC chart

Sample : CDP-ribitol tetra-Moc, diacetate
Sample Size : 1.0 μL(0.63 mg/63 μL-H2O)
Column : XBridge C18 3.5um (4.6 mm I.D. × 150 mm) #01663415523834
Eluent : A Buffer:10 mM AcOH・TEA-H2O
B Buffer:10 mM AcOH・TEA-CH3CN
Gradient : Acetonitrile 10% to 60% [25 min.]
Flow Rate : 1.0 mL/min. , ; Press. : 17 MPa , ; Temp. : 40℃
Detection : CH.1 220 nm

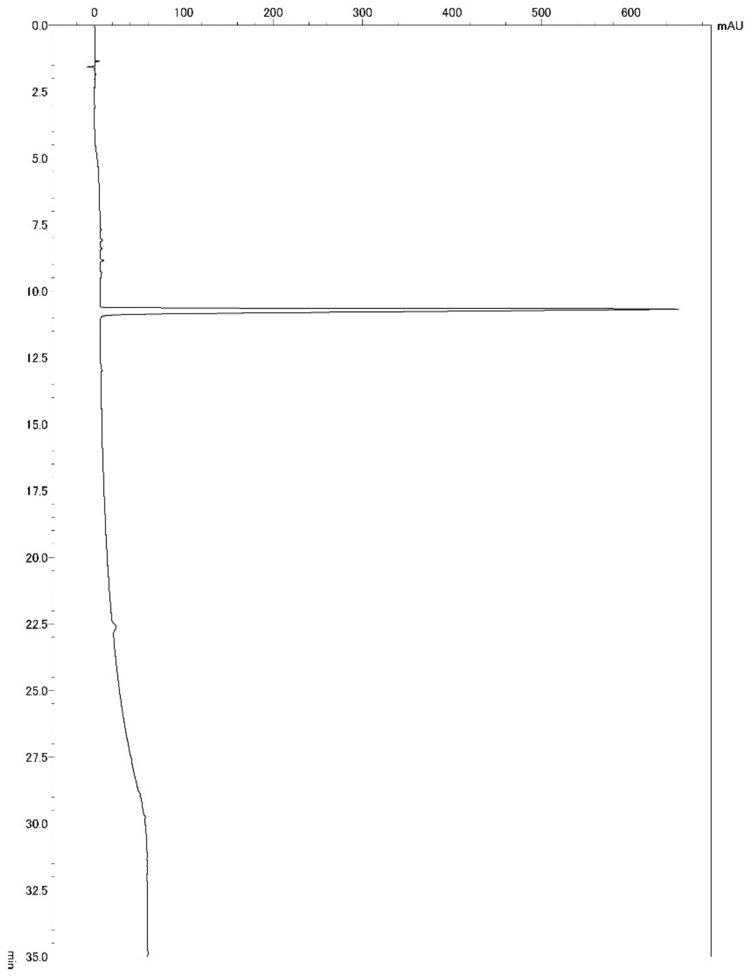

ESI-MS spectrum (negative mode)

$C_{26}H_{36}N_3O_{25}P_2^-$:[M-H]$^-$, calcd: 852.11, found: 852.1

^{1}H-NMR spectrum(400 MHz, in D_2O, ppm)

7.98(d, 1H, J=7.3 Hz), 6.21(d, 1H, J=5.0 Hz), 6.16(d, 1H, J=7.8 Hz), 5.44(dd, 1H, J=4.1 and 5.5 Hz), 5.40(dd, 1H, J=5.5 and 5.5 Hz), 5.29(dd, 1H, J=5.0 and 5.0 Hz), 5.23-5.16(m, 2H), 4.55(dd, 1H, J=3.2 and 12.8 Hz), 4.52(m, 1H), 4.38(dd, 1H, J=6.0 and 12.4 Hz), 4.30(ddd, 1H, J=2.3, 4.1 and 11.9 Hz), 4.23(ddd, 1H, J=3.2, 5.5 and 11.9 Hz), 4.18(ddd, 1H, J=3.2, 4.1 and 11.9 Hz), 4.11(ddd, 1H, J=6.0, 6.0 and 11.9 Hz), 3.82(m, 6H), 3.81(s, 3H), 3.79(s, 3H), 2.17(s, 3H), 2.12(s, 3H)

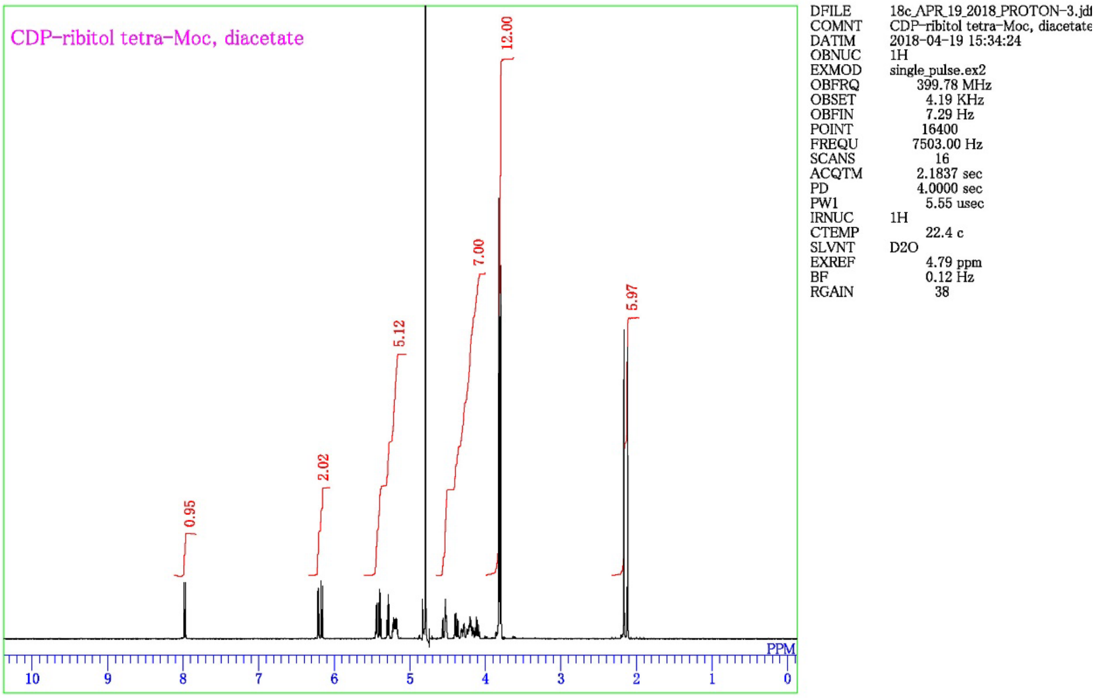

i. CDP-ribitol hexaacetate-PB1 [CDP(DiA)-Rbo(TetA)-PB1]

Chemical Nomenclature

(2S,3S,4R)-5-(((((((2R,3R,4R,5R)-3,4-Diacetoxy-5-(4-amino-2-oxopyrimidin-1(2H)-yl)tetrahydrofuran-2-yl)methoxy)((4-(pentanoyloxy)benzyl)oxy)phosphoryl)oxy)(hydroxy)phosphoryl)oxy)pentane-1,2,3,4-tetrayl tetraacetate

HPLC chart

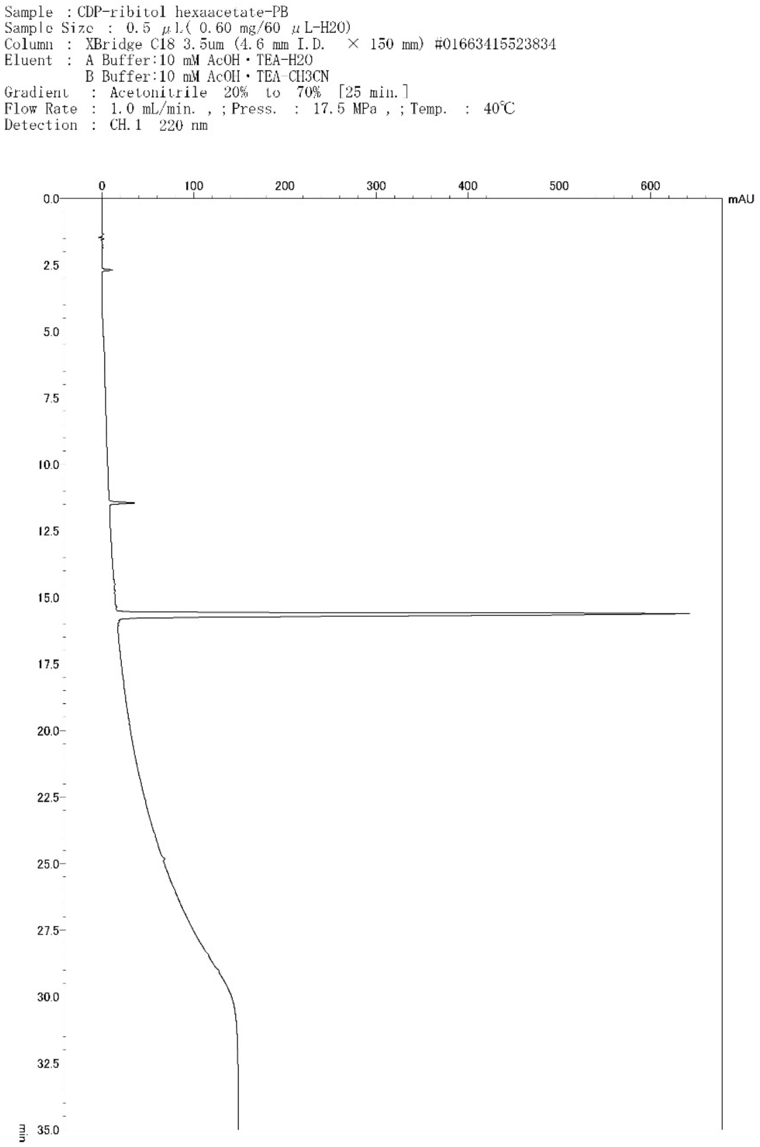

ESI-MS spectrum (negative mode)

$C_{38}H_{50}N_3O_{23}P_2$:[M-H]$^-$, calcd: 978.23, found: 978.2

^{1}H-NMR spectrum(400 MHz, in D_2O, ppm)

7.60(d, 0.5H, J=7.8 Hz), 7.57(d, 0.5H, J=7.3 Hz), 7.56-7.51(m, 2H), 7.18-7.12(m, 2H), 6.10-6.07(m, 1H), 5.97(d, 0.5H, J=7.8 Hz), 5.90(d, 0.5H, J=7.3 Hz), 5.36(dd, 0.5H, J=5.5 and 5.5 Hz), 5.33(dd, 0.5H, J=5.5 and 5.5 Hz), 5.28-5.21(m, 6H), 4.44-4.34(m, 3H), 4.32-4.22(m, 2H), 4.20-4.12(m, 1H), 4.09-4.02(m, 1H), 2.64(t, 1H, J=7.3 Hz), 2.63(t, 1H, J=7.3 Hz), 2.144(s, 3H), 2.122(s, 1.5H), 2.120(s, 1.5H), 2.103(s, 1.5H), 2.098(s, 3H), 2.088(s, 3H), 2.080(s, 1.5H), 2.060(s, 1.5H), 2.057(s, 1.5H), 1.74-1.66(m, 2H), 1.46-1.36(m, 2H), 0.93(t, 3H, J=7.3 Hz)

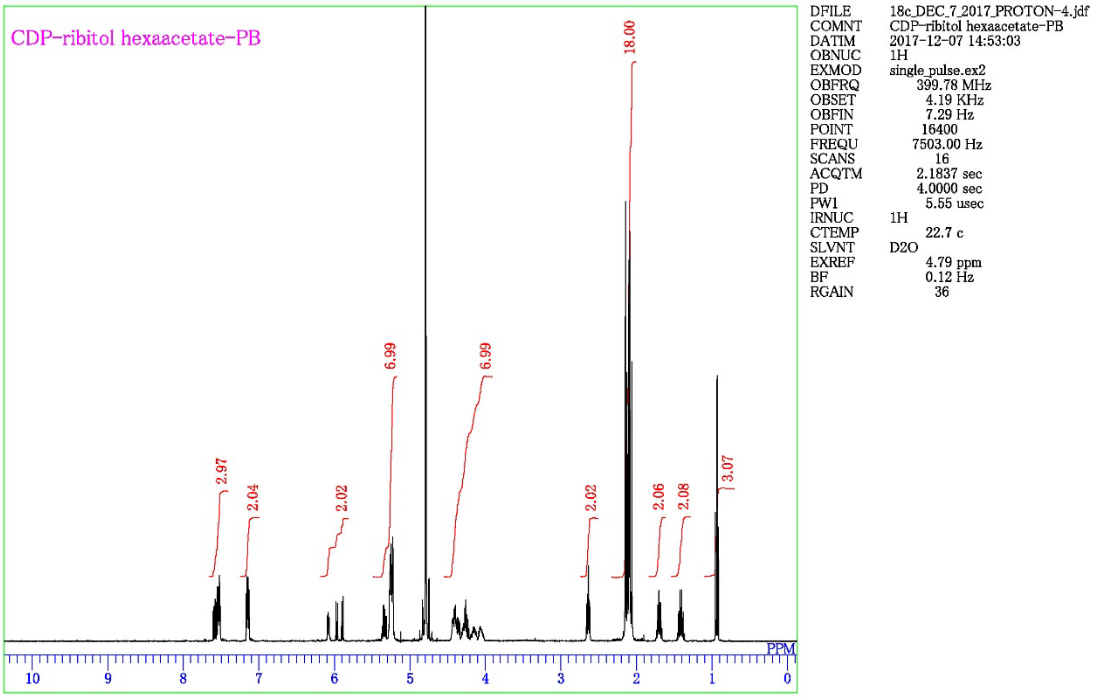

j. CDP-ribitol hexaacetate-PB2 [CDP(DiA)-Rbo(TetA)-PB2]

Chemical Nomenclature

(2S,3S,4R)-5-(((((((2R,3R,4R,5R)-3,4-Diacetoxy-5-(4-amino-2-oxopyrimidin-1(2H)-yl)tetrahydrofuran-2-yl)methoxy)(hydroxy)phosphoryl)oxy)((4-(pentanoyloxy)benzyl)oxy)phosphoryl)oxy)pentane-1,2,3,4-tetrayl tetraacetate

HPLC chart

Sample : CDP-ribitol hexaacetate another-PB
Sample Size : 0.5 μL(3 mg/300 μL-H2O)
Column : XBridge C18 3.5um (4.6 mm I.D. × 150 mm) #01803612413836
Eluent : A Buffer:10 mM AcOH・TEA-H2O
B Buffer:10 mM AcOH・TEA-CH3CN
Gradient : Acetonitrile 20% to 70% [25 min.]
Flow Rate : 1.0 mL/min. , ; Press. : 15.5 MPa , ; Temp. : 40℃
Detection : CH.1 220 nm

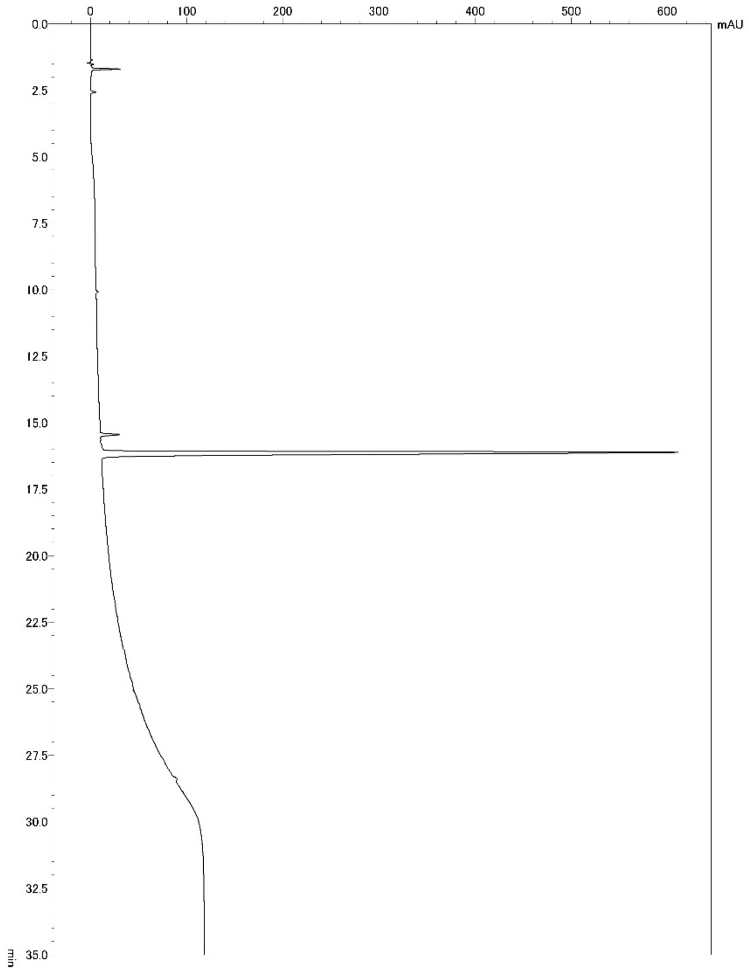

ESI-MS spectrum (negative mode)

$C_{38}H_{50}N_3O_{23}P_2^-$:[M-H]$^-$, calcd: 978.23, found: 978.2

^{1}H-NMR spectrum(400 MHz, in D_2O, ppm)

7.75(d, 0.5H, J=7.8 Hz), 7.73(d, 0.5H, J=7.3 Hz), 7.50(d, 2H, J=8.7 Hz), 7.15(m, 2H), 6.14(m, 1H), 5.99(d, 0.5H, J=7.8 Hz), 5.96(d, 0.5H, J=7.3 Hz), 5.39-5.28(m, 3H), 5.24-5.17(m, 4H), 4.46(m, 1H), 4.37-4.13(m, 6H), 2.64(t, 2H, J=7.3 Hz), 2.155(s, 1.5H), 2.151(s, 1.5H), 2.109(s, 1.5H), 2.104(s, 1.5H), 2.100(m, 3H), 2.084(s, 1.5H), 2.076(m, 4.5 H), 2.06(s, 1.5H), 2.05(s, 1.5H), 1.74-1.67(m, 2H), 1.46-1.37(m, 2H), 0.94(t, 3H, J=7.3 Hz)

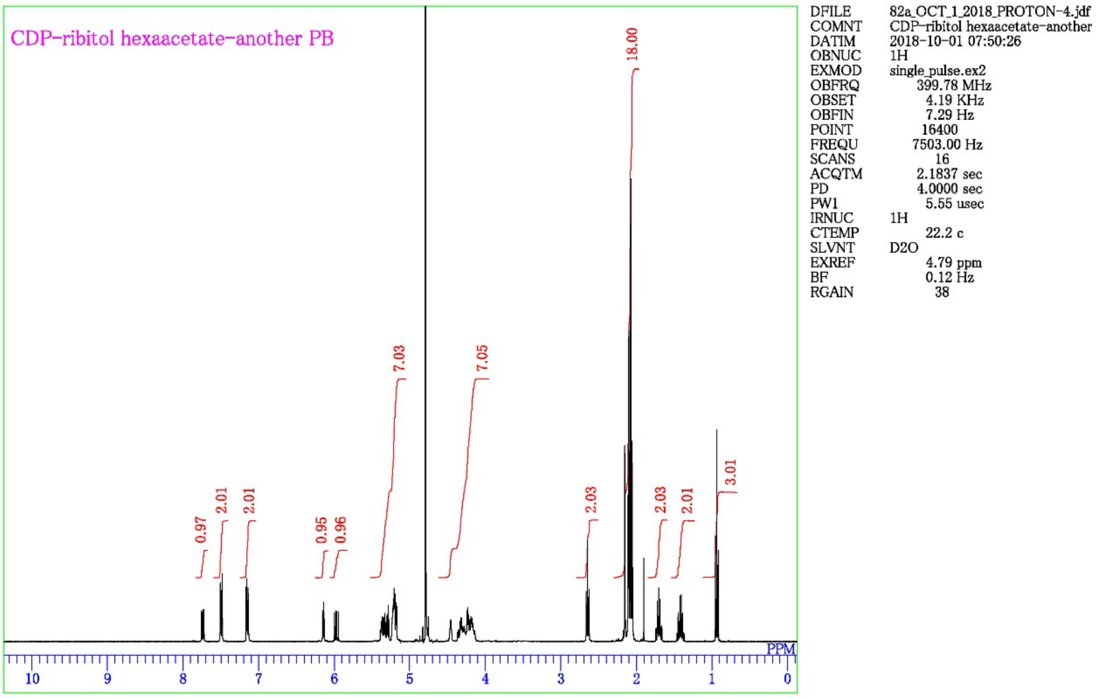

Supplementary Figure 9. Chemical nomenclature, HPLC chart, ESI-MS data, and ^{1}H-NMR spectrum of the synthesized CDP-Rbo derivatives.

Supplementary Table 1: Primer sequences

Primer names	Sequences
Ispd lox forward	5'-CGTATAGCATACATTATACGAAG-3'
Ispd lox reverse	5'-CTTCGTATAATGTATGCTATACG-3'
Myf5 forward	5'-CGTAGACGCCTGAAGAAGGTCAACCA-3'
Myf5 reverse	5'-CACATTAGAAAACCTGCCAACACC-3'
Ispd exon2 forward *(*RT-PCR)	5'-CAGCTACACTTTGCAGGCTATG-3'
Ispd exon2 reverse *(*RT-PCR)	5'-TACAGTCTGGCTGATCTTCTGC-3'
GAPDH forward (RT-PCR)	5'-TCGGTGTGAACGAATTTGGC-3'
GAPDH reverse (RT-PCR)	5'-AATGAAGGGGTCGTTGATGG-3'

Supplementary Table 2: Primary antibodies

Primary antibody names	Provider (Catalog#)	recognizing protein	Dilution
Mouse monoclonal IIH6	Millipore (#05-593)	Glycosylated α-DG (matriglycan)	WB 1:1000, IF 1:50
Rat monoclonal 3D7-7	Ohtsuka et al, 2015	α-DG core	WB 1:1000, IF 1:1000
Mouse monoclonal 8D5	Leica Biosystems (NCL-b-DG)	β-DG	WB 1:2000
Rabbit polyclonal H242	Santa Cruz (sc-23585)	β-DG	WB 1:300, IF 1: 100
Rat monoclonal 4H8-2	Santa Cruz (sc-59854)	Laminin α2	IF 1:200
Rabbit polyclonal L9393	Merck (L9393)	Laminin	Laminin Overlay Assay 1:5000
Rabbit polyclonal ISPD	Abcam (ab107841)	ISPD	WB 1:500
Mouse monoclonal F1.652	The Developmental Studies Hybridoma Bank, University of Iowa (F1.652)	Myosin heavy chain (embryonic)	IF 1:20
Rabbit polyclonal collagen I	Bio-rad (#2150-1440)	Collagen I	IF 1:50
Rat monoclonal F4/80	BioLegend (#123102)	Mouse F4/80 (macrophage)	IF 1:50

Supplementary Table 3: Secondary Antibodies

Secondary antibody names	Provider (Catalog#)	Dilution
Goat anti-mouse-IgM-HRP	Merck (AP128P)	WB: 1:2000
Donkey anti-mouse-IgG HRP	Jackson ImmunoResearch (715-035-150)	WB 1:2000
Rabbit anti-rat-IgG HRP	DAKO (P0450)	WB 1:3000
Goat anti-rabbit-IgG HRP	DAKO (P0448)	WB 1:3000<br>Laminin Overlay Assay 1:5000
Goat anti-rat-IgG<br>Alexa Fluor 488	Thermo Fisher Scientific (A-11006)	IF 1:500
Goat anti-rat-IgG<br>Alexa Fluor 546	Thermo Fisher Scientific (A-11081)	IF 1:500
Goat anti-rabbit-IgG<br>Alexa Fluor 546	Thermo Fisher Scientific (A-11035)	IF 1:500
Goat anti-mouse-IgM<br>Alexa Fluor 488	Thermo Fisher Scientific (A-21042)	IF 1:500
Goat anti-mouse-IgM<br>Alexa Fluor 546	Thermo Fisher Scientific (A-21045)	IF 1:500
Goat anti-mouse-IgG<br>Alexa Fluor 555	Thermo Fisher Scientific (A-21424)	IF 1:500
