# Supplementary material for: CDP-ribitol prodrug treatment ameliorates ISPD-deficient muscular dystrophy mouse model
Source: Nat Commun. 2022 Apr 14;13:1847. doi: 10.1038/s41467-022-29473-4 (PMC9010444; doi:10.1038/s41467-022-29473-4)
Supplement: Supplementary file 2 — Reporting Summary [file 41467_2022_29473_MOESM2_ESM.pdf]

nature portfolio

Corresponding author(s): Motoi Kanagawa
Tatsushi Toda

Last updated by author(s): Jan 28, 2022

Reporting Summary

Nature Portfolio wishes to improve the reproducibility of the work that we publish. This form provides structure for consistency and transparency in reporting. For further information on Nature Portfolio policies, see our Editorial Policies and the Editorial Policy Checklist.

Statistics

For all statistical analyses, confirm that the following items are present in the figure legend, table legend, main text, or Methods section.

n/a	Confirmed	
☐	☒	The exact sample size (*n*) for each experimental group/condition, given as a discrete number and unit of measurement
☐	☒	A statement on whether measurements were taken from distinct samples or whether the same sample was measured repeatedly
☐	☒	The statistical test(s) used AND whether they are one- or two-sided<br>*Only common tests should be described solely by name; describe more complex techniques in the Methods section.*
☒	☐	A description of all covariates tested
☐	☒	A description of any assumptions or corrections, such as tests of normality and adjustment for multiple comparisons
☐	☒	A full description of the statistical parameters including central tendency (e.g. means) or other basic estimates (e.g. regression coefficient) AND variation (e.g. standard deviation) or associated estimates of uncertainty (e.g. confidence intervals)
☒	☐	For null hypothesis testing, the test statistic (e.g. *F*, *t*, *r*) with confidence intervals, effect sizes, degrees of freedom and *P* value noted<br>*Give P values as exact values whenever suitable.*
☒	☐	For Bayesian analysis, information on the choice of priors and Markov chain Monte Carlo settings
☒	☐	For hierarchical and complex designs, identification of the appropriate level for tests and full reporting of outcomes
☒	☐	Estimates of effect sizes (e.g. Cohen's *d*, Pearson's *r*), indicating how they were calculated

Our web collection on statistics for biologists contains articles on many of the points above.

Software and code

Policy information about availability of computer code

Data collection	Histopathological images were collected using fluorescence microscopy (BZ-9000 microscope, Keyence, Osaka, Japan) and analyzed using ImageJ software (https://imagej.nih.gov/ij/). Immunoreactive bands were analyzed using LAS4000 luminescent image analyzer (Fuji Film).
Data analysis	All statistical analyses were performed using GraphPad Prism v.8.20 for Windows (GraphPad Software, San Diego, CA, USA).

For manuscripts utilizing custom algorithms or software that are central to the research but not yet described in published literature, software must be made available to editors and reviewers. We strongly encourage code deposition in a community repository (e.g. GitHub). See the Nature Portfolio guidelines for submitting code & software for further information.

Data

Policy information about availability of data

All manuscripts must include a data availability statement. This statement should provide the following information, where applicable:

- Accession codes, unique identifiers, or web links for publicly available datasets
- A description of any restrictions on data availability
- For clinical datasets or third party data, please ensure that the statement adheres to our policy

The data that support the findings of this study are available from the corresponding author upon reasonable request.

Field-specific reporting

Please select the one below that is the best fit for your research. If you are not sure, read the appropriate sections before making your selection.

- [x] Life sciences
- [ ] Behavioural & social sciences
- [ ] Ecological, evolutionary & environmental sciences

For a reference copy of the document with all sections, see nature.com/documents/nr-reporting-summary-flat.pdf

Life sciences study design

All studies must disclose on these points even when the disclosure is negative.

Sample size	No statistical methods were used to predetermine sample sizes. Sample sizes were chosen to determine whether or not the results were statistically significant.
Data exclusions	No data exclusion
Replication	Either 3 or more times. All attempts at replication were successful.
Randomization	Mice were allocated into three genotyping groups, WT control, heterozygous control, and cKO. Treatments of cKO mice were randomly allocated into groups.
Blinding	Immunofluorescence and biochemical analyses were blinded.

Reporting for specific materials, systems and methods

We require information from authors about some types of materials, experimental systems and methods used in many studies. Here, indicate whether each material, system or method listed is relevant to your study. If you are not sure if a list item applies to your research, read the appropriate section before selecting a response.

Materials & experimental systems

n/a	Involved in the study	
[ ]	[x]	Antibodies
[ ]	[x]	Eukaryotic cell lines
[x]	[ ]	Palaeontology and archaeology
[ ]	[x]	Animals and other organisms
[x]	[ ]	Human research participants
[x]	[ ]	Clinical data
[x]	[ ]	Dual use research of concern

Methods

n/a	Involved in the study	
[x]	[ ]	ChIP-seq
[x]	[ ]	Flow cytometry
[x]	[ ]	MRI-based neuroimaging

Antibodies

Antibodies used

Glycosylated α-DG, Mouse monoclonal IIH6, Millipore (#05-593)
α-DG core, Rat monoclonal 3D7-7, Ohtsuka et al, 2015
β-DG, Mouse monoclonal 8D5, Leica Biosystems (NCL-b-DG)
β-DG, Rabbit polyclonal H242, Santa Cruz (sc-23585)
Laminin α2, Rat monoclonal 4H8-2, Santa Cruz (sc-59854)
Laminin, Rabbit polyclonal L9393, Merck (L9393)
ISPD, Rabbit polyclonal ISPD, Abcam (ab107841)
Myosin heavy chain (embryonic), Mouse monoclonal F1.652, The Developmental Studies Hybridoma Bank, University of Iowa (F1.652)
Collagen I, Rabbit polyclonal collagen I, Bio-rad (#2150-1440)
Mouse F4/80 (macrophage), Rat monoclonal F4/80, BioLegend (#123102)
Goat anti-mouse-IgM-HRP, Merck (AP128P)
Donkey anti-mouse-IgG HRP, Jackson ImmunoResearch (715-035-150)
Rabbit anti-rat-IgG HRP, DAKO (P0450)
Goat anti-rabbit-IgG HRP, DAKO (P0448)
Goat anti-rat-IgG Alexa Fluor 488, Thermo Fisher Scientific (A-11006)
Goat anti-rat-IgG Alexa Fluor 546, Thermo Fisher Scientific (A-11081)
Goat anti-rabbit-IgG Alexa Fluor 546, Thermo Fisher Scientific (A-11035)
Goat anti-mouse-IgM Alexa Fluor 488, Thermo Fisher Scientific (A-21042)
Goat anti-mouse-IgM Alexa Fluor 546, Thermo Fisher Scientific (A-21045)
Goat anti-mouse-IgG Alexa Fluor 555, Thermo Fisher Scientific (A-21424)

Validation	All antibodies were validated by the supplier. We also used negative controls in each experiment.

Eukaryotic cell lines

Policy information about cell lines

Cell line source(s)	HEK293 cells were obtained from ATCC. ISPD-deficient HEK293 cells were described previously (Kanagawa et al, Cell Reports 2016)
Authentication	Lines were confirmed by direct sequencing.
Mycoplasma contamination	Cell lines were negative for Mycoplasma.
Commonly misidentified lines (See ICLAC register)	No commonly miss identified lines.

Animals and other organisms

Policy information about studies involving animals; ARRIVE guidelines recommended for reporting animal research

Laboratory animals	Heterozygous Ispd flox mice (Ispdlox/+) were generated by the mouse biology program (MBP) at the University of California, Davis (C57BL/6N-Ispdem2Mbp/Mmucd; RRID: MMRRC_037583-UCD) and were intercrossed to obtain homozygous floxed mice (Ispdlox/lox). Myf5-Cre knock-in (KI) mice (Myf5-CreKI (+)) obtained from The Jackson Laboratory (B6.129S4-Myf5tm3(cre)Sor/J; Stock No 007893) were backcrossed for more than six generations with C57BL/6 mice before crossing with Ispdlox/lox mice. Heterozygous Ispdlox/+ mice carrying Myf5-Cre [Ispdlox/+: Myf5-CreKI (+)] were bred with Ispdlox/lox mice to obtain Myf5-Ispd-cKO mice. We used both male and female mice for this study. Mice were analyzed at 4 – 20 weeks of age.
Wild animals	This study did not involve wild animals.
Field-collected samples	This study did not involve samples collected from the field.
Ethics oversight	All animal experiments were approved by the Animal Care and Use Committees of Kobe University Graduate School of Medicine (P150605, P180901, and P200409) and Ehime University Graduate School of Medicine (05-O-70-1).

Note that full information on the approval of the study protocol must also be provided in the manuscript.
